# Multi-omics profiling reveals divergent biology and liver microenvironment in HCC of metastatic and *de novo* origin

**DOI:** 10.1186/s12943-026-02604-x

**Published:** 2026-03-06

**Authors:** Gina F. Boot, Fabian Haak, Mairene Coto-Llerena, Tuyana Boldanova, Eva Dazert, Philip Sedlaczek, George Rosenberger, Cinzia Esposito, Caner Ercan, Stefan Wieland, Salvatore Lorenzo Renne, Andrej Benjak, Matthias S. Matter, Luca Di Tommaso, Michael N. Hall, Luigi M. Terracciano, Markus H. Heim, Salvatore Piscuoglio, Charlotte K. Y. Ng

**Affiliations:** 1 Department of Biomedicine, University of Basel, Basel, Switzerland; 2 Institute of Medical Genetics and Pathology, University Hospital Basel, Basel, Switzerland; 3 University Digestive Health Care Center Basel – Clarunis, Basel, Switzerland; 4 Department of Visceral, Transplant, Thoracic and Vascular Surgery, University Hospital of Leipzig, Leipzig, Germany; 5 Biozentrum, University of Basel, Basel, Switzerland; 6 Department of Biomedical Sciences, Humanitas University, Via Rita Levi Montalcini 4, Pieve Emanuele, 20072 Milan, Italy; 7 IRCCS Humanitas Research Hospital, Rozzano, Milan Italy; 8 Department for BioMedical Research (DBMR), University of Bern, Bern, Switzerland

**Keywords:** Hepatocellular carcinoma, Multicentric occurrence, Intrahepatic metastasis, Multiomics profiling, Proteomics

## Abstract

**Background:**

Multifocal hepatocellular carcinoma (mfHCC) arises via intrahepatic metastasis (IM) or multicentric occurrence (MO), each with distinct biological behavior and clinical implications, though mfHCC origin is rarely assessed in clinical practice. We aimed to characterize the clinicopathological and molecular features of IM-HCC, MO-HCC, and their surrounding non-tumor liver (NTL) tissues using multi-omics analyses.

**Methods:**

We analyzed 76 tumor and 44 NTL biopsies from 22 patients, using whole-exome sequencing, RNA-sequencing, and proteomic/phosphoproteomic profiling. Patients were classified as IM, MO or mixed (IM + MO) according to their somatic mutations. A comparator cohort of 48 unifocal HCC and 15 normal livers was used. Clinicopathological parameters, pathway and transcription factor activities, immune infiltration, and targetable alterations were assessed.

**Results:**

Clonality analysis identified 10 IM, 9 MO, and 3 mixed patients. IM-HCCs showed more frequent macrovascular invasion and extrahepatic metastases, with upregulation of E2F/MYC-related cell cycle pathways, higher expression of metastasis-associated genes (e.g., *TTK*, *BUB1*, *NUF2*), higher CD8 + T-cell exhaustion, and shared actionable mutations (e.g. *PTEN*). MO-HCCs within patients displayed molecular dissimilarity comparable to tumors from different patients, though they also showed convergent kinase and pathway dysregulation. NTLs of IM-HCC patients had lower fibrosis, extracellular matrix signaling and pro-regenerative pathways (e.g., *SOX2*, *TGFA*) than those of MO-HCC.

**Conclusions:**

The aggressive molecular features and immune exhaustion of IM-HCC support the need for combined therapies, while the convergence of kinase and pathway dysregulation of MO-HCC provides unified therapeutic opportunities. Key differences in fibrogenic and regenerative pathways may influence metastatic potential. Our findings provide insights into the biological behavior and therapeutic opportunities in mfHCC.

**Supplementary Information:**

The online version contains supplementary material available at 10.1186/s12943-026-02604-x.

## Background

Hepatocellular carcinoma (HCC) typically arises in the context of chronic liver inflammation, an environment conducive to tumorigenesis. HCC multifocality, with 30–40% of patients presenting with multiple tumor nodules [1], often renders these patients unresectable [2]. HCC multifocality arises through two distinct mechanisms: intrahepatic metastases (IM) and multicentric occurrence (MO). IM-HCC originates from disseminated tumor cells with a common clonal origin, while MO-HCC represents *de novo* hepatocarcinogenesis of independent lesions, involving multiple regenerative nodules acting as tumor initiation sites [3] in a damaged liver driven by the pro-oncogenic ‘cancer field effect’ [4]. IM-HCCs are more genomically unstable and aggressive, and are associated with AFP elevation and vascular invasion [5, 6]. IM-HCCs also show worse survival outcomes and earlier recurrence, whereas MO-HCCs tend to respond better to regional therapy and have better (postoperative) survival outcomes [7–9].

Despite the high prevalence of multifocal HCC (mfHCC) and the divergent outcomes, mfHCC origins are not routinely distinguished. Earlier research studies primarily used clinical [10] and histopathological [9, 11] criteria to distinguish the entities. Genomic analysis has enabled a more accurate distinction [5, 12–14], and has been combined with transcriptomics [13, 15], epigenomics [5], metabolomics [16], and immunopeptidomics and T cell receptor sequencing [17] to provide insights into tumor clonality, immune landscape, and therapeutic potential. These studies have revealed the high expression of the mitotic checkpoint kinase *TTK* and metastasis-related genes (*AGR2*, *HOXB9*, *CEACAM6*) and enrichment of pathways including epithelial-to-mesenchymal transition, G2/M, and E2F signaling among IM-HCCs [13, 16]. Immunologically, IM-HCCs had reduced T-cell infiltration whereas MO-HCCs displayed increased immune checkpoint expression, which could influence response to immunotherapy [17]. Consistently, single-cell RNA-sequencing has revealed that terminally exhausted and regulatory T cell niches are more prevalent in IM-HCCs [18].

The non-tumor liver (NTL) in HCC patients is not a bystander in hepatocarcinogenesis. The fibrotic and cirrhotic environment is prone to epigenetic reprogramming, immune modulation and regenerative stress [19, 20]. Activation of hepatic stellate cells, as well as dysregulation in immune and other signaling pathways such as NF-κB, TGF-β, WNT and HNF4a, all contribute to the transition to HCC [19, 20]. Multi-omics profiling also supports the ‘field effect’ theory, where an epigenetically and transcriptionally primed liver environment supports tumor emergence [21]. While fibrosis is generally considered pro-tumorigenic (enhancing angiogenesis, extracellular matrix (ECM) remodeling and inflammatory signaling), its role in metastasis is nuanced. Fibrosis might impede tumor spread by creating physical, fibrous barriers; however, most evidence supports a metastasis-promoting role through the actions of activated hepatic stellate cells and immune-suppressive signaling in the local tumor microenvironment, but less so in the adjacent liver [22].

Here we investigate a unique cohort of 22 mfHCC patients with multiomics profiling performed on distinct tumor nodules. Using genomic, transcriptomic and (phospho)proteomic analyses, this study aims to evaluate tumor heterogeneity, biological underpinnings and therapeutic opportunities in the distinct origins of mfHCC, while also exploring the molecular features of the NTL to understand how hepatic pathology may contribute to HCC development.

## Methods

### Patients and tissue sample

Tissues were obtained from 22 HCC patients with at least two distinct tumor nodules undergoing liver biopsies at the University Hospital Basel between 2008 and 2018 (Additional file 2). Written informed consent was obtained from all patients to use additional biopsy material from diagnostic procedures for research purposes. The study was approved by the ethics committee of the northwestern part of Switzerland (EKNZ 2014–099). Ultrasound-guided needle biopsies were obtained from tumors and the liver parenchyma at a site distant from the tumors (NTL) as described [23]. The number and timing of the biopsies depended on the size and number of tumor nodules. An expert radiology team evaluated computed tomography scans acquired prior to biopsy according to RECIST guidelines [24], confirming 19 had clinically determined multifocal disease at diagnosis.

These 22 patients are a subset of our recent multi-omics cohort [23] and all patients had at least one tumor biopsy at diagnosis. From the 22 patients, 76 HCC and 44 NTL biopsies were included (Additional file 3). None of the patients had received systemic or locoregional therapies for HCC prior to the initial diagnostic biopsy. Clinical staging was performed using the Barcelona Clinic Liver Cancer (BCLC) system [25]. For tumor location, Couinaud segments sharing a common boundary, like a hepatic vein or a portal triad, are considered neighboring, and contralateral lobe means left vs right lobe. See Supplementary Methods for details regarding longitudinal tissue collection and histopathological assessment. We further included the clinicopathological parameters of 44 patients with mfHCC at diagnosis but did not have biopsies from multiple nodules [23]. As comparison groups, we included data from 48 HCC and 48 NTL samples from 48 patients with unifocal (single nodule) HCC, and liver biopsies with normal histology from 15 patients without HCC and with normal liver values [23].

### Multiomics profiling and data analysis

Whole-exome and RNA-sequencing data for the 76 HCC and 44 NTL biopsies of the 22 mfHCC patients were newly generated for this study or retrieved from [23, 26]. Whole-exome and RNA-sequencing data for HCCs and NTLs of 48 unifocal HCC patients were retrieved from [23]. (Phospho)proteome data for 22 HCC and 16 NTL biopsies from 9 mfHCC patients, as well as all HCCs and NTLs from 22 unifocal HCC patients were retrieved from [23, 26]. Two unifocal HCC patients did not have phosphoproteomic data. RNA-sequencing and (phospho)proteome data were retrieved for 15 and 5 normal livers, respectively [23]. Further details in Supplementary Methods and Additional file 3.

### Analysis of clonal relationship

BreakClone [27] was used to assess the clonal relationships between tumor pairs based on their somatic single nucleotide variants and small insertions and deletions (Supplementary Methods). Patients whose tumor nodules were all clonal or all non-clonal to one another were defined as IM and MO patients, respectively. Patients with tumor nodules of both clonal and non-clonal relationships were defined as mixed IM + MO patients.

### Comparison of TMP and TNEM molecular profiles

A tumor with metastatic potential (TMP) was defined as a set of clonally related tumors, while a tumor with no evidence of metastasis (TNEM) was defined as a single tumor not clonally related to any other tumor. For the comparisons of mutational frequencies between TMP and TNEM, the union of mutations of the biopsies were used per TMP/TNEM. For the comparisons of frequencies of copy number gains and losses between TMP and TNEM, the maximum absolute copy number states of the biopsies were used per TMP/TNEM. For other parameters, including tumor mutational burden (TMB), the average abundance of markers or enrichment of pathways, the first biopsy of individual nodules per TMP/TNEM was used, unless otherwise indicated. For clinicopathological variables, the status of the first nodule biopsied of the TMP/TNEM was used.

### Statistical analysis

Correlation analyses were performed using Spearman's correlation. Statistical comparisons of clinicopathological variables were performed using Mann–Whitney U tests, Fisher’s exact tests or chi-squared tests, or Kruskal–Wallis tests. All statistical tests were two-sided. p ≤ 0.05 was considered statistically significant. In all figures, ****: *p* < 0.0001, ***: *p* < 0.001, **: *p* < 0.01, * *p* < 0.05, ns: not significant. See Supplementary Methods for details.

## Results

### IM-HCC patients demonstrate clinicopathological features of more aggressive disease

Our previously published cohort of 114 HCC patients [23] comprised unifocal and synchronous mfHCC patients, defined respectively as patients with one or at least two tumor nodules at diagnosis. Compared to unifocal patients (*n* = 51, 45%), patients with synchronous mfHCC (*n* = 63, 55%) were older, had higher AFP levels and had shorter overall survival, but there was no difference in the presence of cirrhosis at diagnosis (Supplementary Fig. 1a and Additional file 4).

**Fig. 1:**
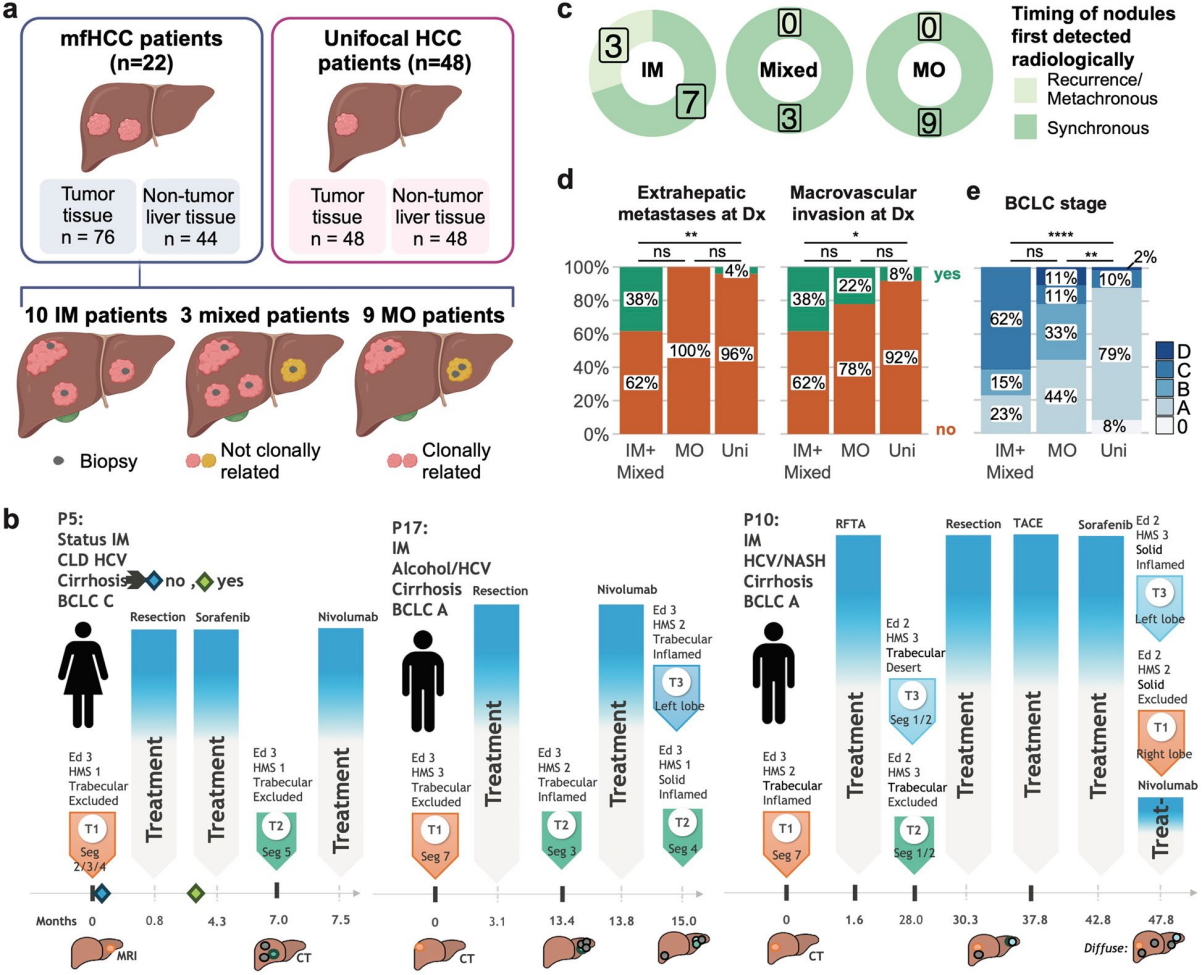
IM-HCC patients demonstrate clinicopathological features of more aggressive disease. **a** Study cohort and the mfHCC classification based on the clonal relatedness analysis of tumor pairs. Figure made with Biorender. **b** Clinical course of the three patients who presented with recurrence. **c** Pie charts illustrating the number of patients whose tumor nodules presented synchronously at diagnosis or as recurrence (i.e. metachronously). **d**-**e** Stacked bar plots illustrating the percentage of patients with (**d**) extrahepatic metastases and macrovascular invasion and (**e**) BCLC stage, stratified into IM/mixed-HCC, MO-HCC and unifocal HCC patients. IM: *n* = 10, mixed: *n* = 3, MO: *n* = 9, unifocal HCC: *n* = 48. Statistical comparisons were performed by (**d**) Fisher's exact tests and (**e**) Mann–Whitney U tests. ****: *p* < 0.0001, ***: *p* < 0.001, **: *p* < 0.01, * *p* < 0.05, ns: not significant

We identified a subset of 22 patients from whom biopsies were obtained from at least two distinct tumor nodules in the liver at various timepoints and represented the major etiologies underlying HCC (Additional file 2). In total, 76 tumor biopsies (median 2 biopsies per patient (range 2–9) derived from 2 nodules per patient (range 2–4)) and 44 NTL biopsies were subjected to multiomics profiling (Additional file 3). To determine the clonal relationship between tumor biopsy pairs, we computed the BreakClone scores [27] based on the somatic mutations (Methods). Of the 110 tumor biopsy pairs derived from samples from distinct tumor nodules, we found 75 clonal and 35 non-clonal pairs (Additional file 5). Overall, we identified 10 IM-HCC patients with only clonal tumor pairs, 9 MO-HCC patients with only non-clonal tumor pairs and 3 mixed patients with both clonal and non-clonal tumor pairs (Fig. 1a). For comparison, a reference cohort of 48 patients with unifocal HCC with single tumor biopsies and matched NTL samples was included and analyzed.

From a clinical standpoint, 18 had synchronous mfHCC at diagnosis, 1 had synchronous mfHCC at their second HCC diagnosis and 3 had unifocal HCC at diagnosis and developed further tumor nodules on recurrence. The median interval between the first and last biopsy was 4 months (range 0–6.9 years). All 3 patients with unifocal HCC at diagnosis were IM-HCC, including P10, whose late recurrence occurred after > 28 months. These three patients had systemic (P5), surgical (P17) and locoregional (P10) therapies prior to recurrence (Fig. 1b). We extensively reviewed the radiology data and found that, except for these 3 patients, all biopsied tumor nodules were already present at diagnosis (Fig. 1c).

We compared the clinicopathological parameters in IM-HCC (IM/mixed), MO-HCC, and unifocal HCC patients. Extrahepatic metastasis and macrovascular invasion at diagnosis were significantly more common in IM/mixed than unifocal patients (38% vs 4% and 38% vs 8%), whereas there was a numerical increase toward higher frequencies of both features in IM/mixed compared to MO patients (38% vs 0% and 38% vs 22%; Fig. 1d and Additional file 6), though the these differences did not reach statistical significance. IM/mixed patients also developed extrahepatic metastases more often during their clinical course than MO (77% vs 11%; Supplementary Fig. 1b-c) and showed a trend toward higher BCLC stage (Fig. 1e). There was a slightly higher number of discrete tumors biopsied, but not the total number of biopsy samples taken, in the IM/mixed compared to MO patients, despite no difference in the number of tumors reported at initial diagnosis (Supplementary Fig. 1 d).

Our findings indicate that patients with IM-HCC (*n* = 10) and MO-HCC (*n* = 9) are similarly frequent among mfHCC. IM-HCC patients showed a higher burden of adverse clinical features, including more advanced BCLC stage and higher frequencies of extrahepatic metastases and macrovascular invasion; these differences were statistically significant when compared with unifocal HCC patients, but represented non-significant trends when compared with MO-HCC, which likely reflects the limited size of the mfHCC patient subgroups.

### Clonally unrelated tumor nodules exhibit a high degree of heterogeneity

While IM tumors may be expected to share molecular and pathological similarities, we sought to investigate the genomic, transcriptomic and histopathological (dis)similarities in MO tumors. We quantified the similarities between clonal and non-clonal pairs of tumor biopsy and compared them to random biopsy pairs from different patients ("inter-patient").

**Fig. 2:**
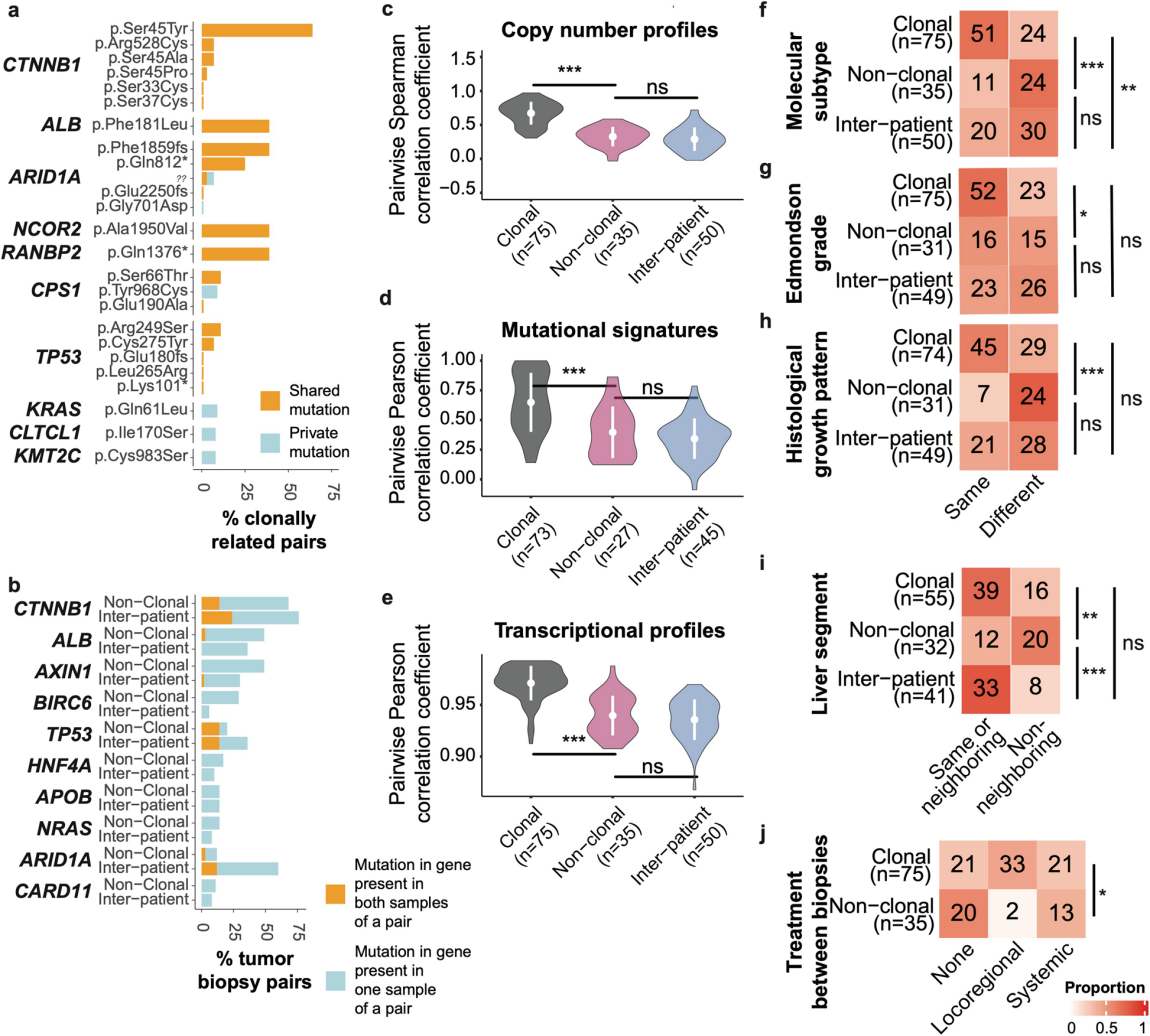
Clonally unrelated tumor nodules exhibit a high degree of heterogeneity. **a** Barplots illustrate the % of clonally related pairs (*n* = 75) with specific shared and private mutations in HCC driver genes. **b** Barplots illustrate the % of non-clonal pairs (*n* = 35) and inter-patient pairs (*n* = 50) with mutations in HCC driver genes in one or both samples of a pair. **c**-**e** Violin plots illustrating the pairwise Spearman/Pearson correlations between the (**c**) copy number profiles, (**d**) mutational signature activities and (**e**) transcriptional profiles of clonal, non-clonal, and random inter-patient biopsy pairs. **f**-**i** Heatmaps of (**f**) Hoshida molecular subtypes, (**g**) Edmondson grade and (**h**) histological growth pattern and (**i**) liver segment of the biopsy between clonal, non-clonal and random inter-patient biopsy pairs. **j** Heatmap illustrating the treatment between biopsies between clonal, non-clonal biopsy pairs. Statistical comparisons were performed by (**c**-**e**) Mann–Whitney U tests, (**f**-**i**) Fisher's exact tests and (**j**) a chi-squared test. ****: *p* < 0.0001, ***: *p* < 0.001, **: *p* < 0.01, * *p* < 0.05, ns: not significant

Genomically, 83% of the mutations in HCC driver genes were shared between clonal pairs (Fig. 2a). *CTNNB1*, *ALB* and *TP53* mutations were always shared. While non-clonal pairs did not share identical mutations in these genes, occasionally, both biopsies had different mutations in the same gene, predominantly in *CTNNB1* and *TP53* (Fig. 2b). However, the frequency of these co-occurring mutations did not differ from that of inter-patient random tumor pairs. Clonal pairs also had higher similarity in terms of copy number, mutational signatures, transcriptomic profiles and molecular subtypes than non-clonal pairs (Fig. 2c-f). Importantly, non-clonal pairs were not more similar to each other than random inter-patient pairs.

Evaluating the clinicopathological features, clonal pairs more frequently had the same histological (Edmondson) grade and major histological growth pattern than non-clonal pairs, while non-clonal pairs did not differ from random inter-patient pairs (Fig. 2g-h and Supplementary Fig. 2a-b). No difference was observed in terms of immunophenotype. Compared to non-clonal pairs, clonal pairs were more likely to arise in the same or neighboring liver segment and more often had intervening locoregional treatment (Fig. 2i-j), but there was no difference for time elapsed between biopsies being taken (Supplementary Fig. 2c). As some tumor nodules were biopsied multiple times, we repeated the analyses using only the first (i.e. first time point) biopsy of each tumor nodule and obtained similar results (Supplementary Fig. 2d-g).

Overall, our comparisons between tumor pairs demonstrated that molecular features are preserved during intrahepatic dissemination but diverge during multicentric tumorigenesis. Crucially, our results also demonstrate that clonally unrelated tumors display a high degree of molecular and histopathological heterogeneity, the extent of which is similar to that of tumors from different individuals.

### Molecular signatures underlying tumors with intrahepatic metastasis

**Fig. 3:**
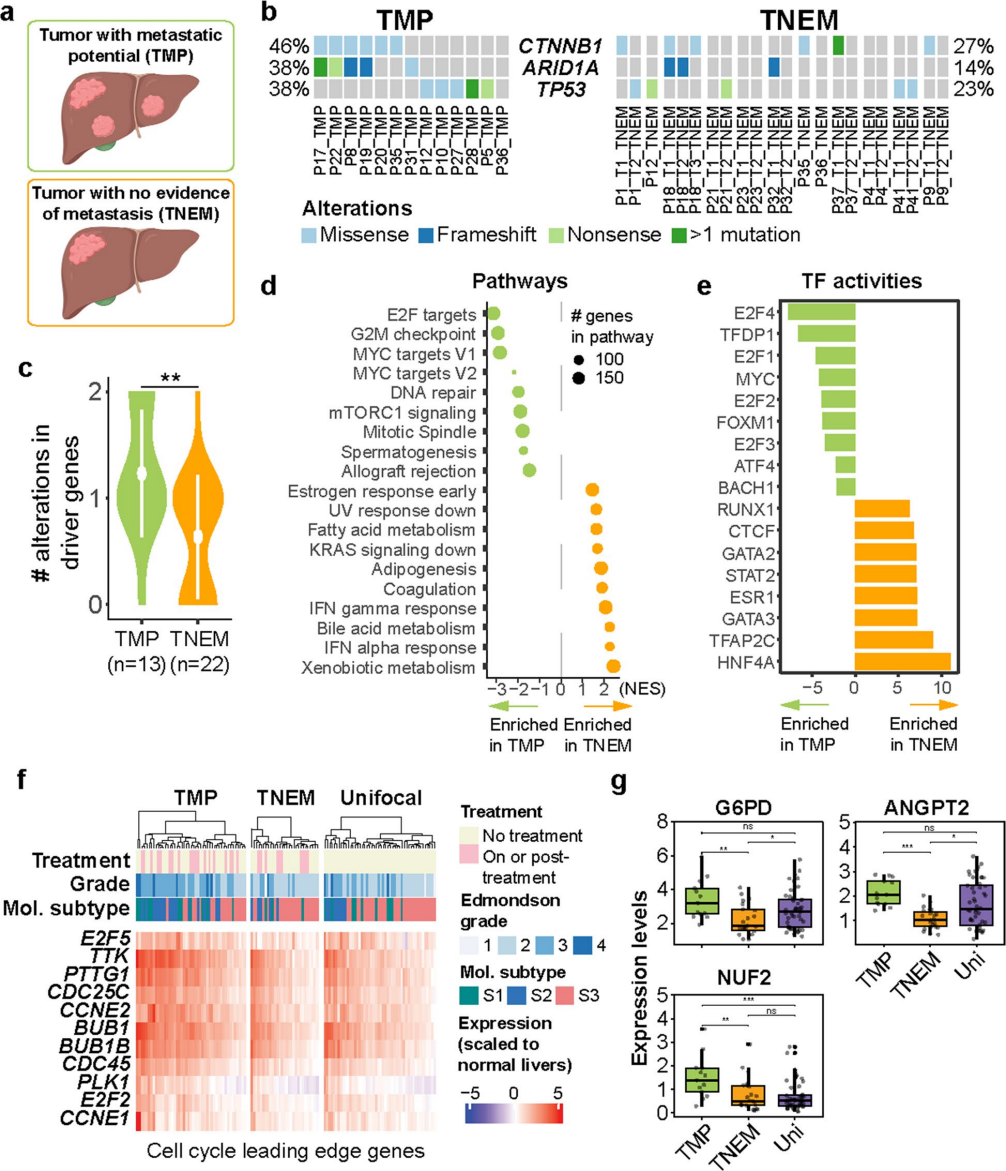
Molecular signatures underlying tumors with intrahepatic metastasis. **a** Illustration of the definitions of tumor with metastatic potential (TMP) and tumor with no evidence of metastasis (TNEM). Figure made with Biorender. **b** Somatic genetic alterations in HCC driver genes, with the types of alterations color-coded according to the color key. **c** Violin plot of the number of alterations in HCC driver genes in (**b**). **d** Bubble plot of the normalized enrichment score (NES) from gene set enrichment analysis of differential expression (transcriptomic) between TMP and TNEM. **e** Barplot of the regulatory activity (enrichment score) of the transcription factors enriched in TMP or TNEM. **f** Heatmap of the expression of the leading edge genes of the KEGG cell cycle pathway, stratified into TMP, TNEM and unifocal HCC. **g** Expression of *G6PD*, *NUF2* and *ANGPT2* as log2(TPM counts). **c**, **g** Statistical comparisons were performed by Mann–Whitney U tests. ****: *p* < 0.0001, ***: *p* < 0.001, **: *p* < 0.01, * *p* < 0.05, ns: not significant

Next, we sought to identify the molecular signatures of metastasis by comparing tumors that did and did not metastasize. Here we considered a tumor with metastatic potential (TMP) as a set of clonally related tumors, i.e. tumors that have metastasized intrahepatically (Fig. 3a and Methods). On average, a TMP consists of the biopsies from 2.5 tumor nodules (range 2–4). Meanwhile, a tumor with no evidence of metastasis (TNEM) refers to a single tumor not clonally related to any other tumor. IM patients, by definition, had 1 TMP where all their tumors were clonally related, whereas the 3 mixed patients each had 1 TMP and 1 TNEM. The 8 MO patients had 2 TNEMs each and 1 patient had 3.

Between TMPs and TNEMs, the mutational frequencies of individual HCC driver genes *CTNNB1*, *ARID1A* and *TP53* did not significantly differ (Fig. 3b). However, collectively, TMPs harbored more frequent alterations in these three genes than TNEMs (Fig. 3c). There was little difference between mutational signature activities, indicating that the underlying mutagenic processes that shaped the cancer genomes did not differ between TMPs and TNEMs (Supplementary Fig. 3a). TMPs had slightly elevated genomic instability in terms of the overall genomic instability (as measured by the fraction of genome altered by copy number alterations) than TNEMs, but not in terms of the frequencies of copy number gains and losses at specific loci (Supplementary Fig. 3b-c).

While TMPs and TNEMs did not fully segregate in terms of their molecular profiles (Supplementary Fig. 3d-f), we identified differentially expressed pathways and transcription factor activities. TMPs showed overexpression of cell cycle-related genes and pathways. These include E2F and MYC targets, G2M checkpoints and mitotic spindle and, correspondingly, increased transcription factor activities of the E2F family, MYC and FOXM1 (Fig. 3d-f and Supplementary Fig. 3g-j). *G6PD, NUF2* and *ANGPT2,* implicated in early intrahepatic recurrence or metastasis [28–30], were also upregulated in TMPs (Fig. 3g). By contrast, TNEMs showed enrichment of interferon signaling and metabolic pathways, as well as HNF4A and GATA3 transcription factor activity, suggesting a more metabolically functional profile (Fig. 3d-e and Supplementary Fig. 3g). Accordingly, TNEMs exhibited a lower Edmondson grade than TMPs and were more frequently characterized as the nonproliferative molecular subtype (Supplementary Fig. 3l-m).

Taken together, TMPs demonstrated enrichment of cell cycle pathways and higher expression of progression-related genes, in accordance with their aggressive histopathological features.

### Identifying targetable avenues across tumor nodules

**Fig. 4:**
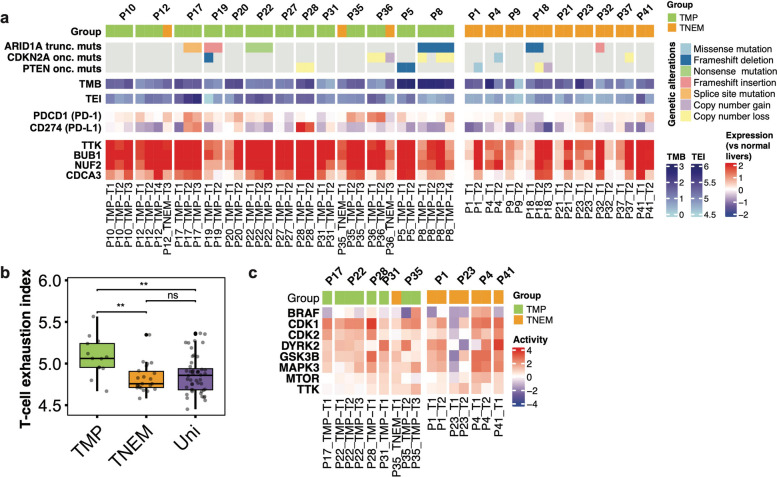
Identifying targetable avenues across tumor nodules. **a** Actionable somatic genetic alterations, tumor mutational burden (TMB), T-cell exhaustion index (TEI), expression levels of immune checkpoint genes PD-1 and PD-L1, and cell cycle genes (TTK, BUB1, NUF2 and CDCA3). **b** Boxplot of T-cell exhaustion index stratified into TMP, TNEM and unifocal HCC. **c** Heatmap of kinase activities as inferred by Kinase Substrate Enrichment Analysis. **b** Statistical comparisons were performed by Mann–Whitney U tests. ****: *p* < 0.0001, ***: *p* < 0.001, **: *p* < 0.01, * *p* < 0.05, ns: not significant

Multifocal HCC poses a clinical challenge because treatment decisions are often based on limited sampling despite underlying molecular diversity. We therefore investigated whether comprehensive genetic and signaling profiling across tumor nodules could identify clinically actionable vulnerabilities at the patient level. According to OncoKB, genes with mutations with compelling biological evidence for drug response (Level 4) included *ARID1A*, *PTEN* and *CDKN2A* (Fig. 4a). In IM-HCC patients, we identified *ARID1A* truncating mutations, targetable by Tazemetostat or PLX2853, shared across all tumor nodules in 3 patients, and *PTEN* oncogenic mutations, targetable by GSK2636771 or AZD8186, in 2 patients. These findings indicate that, despite the multifocal nature of the disease, a subset of IM-HCC patients harbor targetable alterations across all biopsied tumor nodules. By contrast, *CDKN2A* alterations, targetable by cell cycle inhibitors such as Palbociclib, were heterogeneous in 3 patients, highlighting that not all actionable events are uniformly shared. In MO-HCC patients, alterations in all three genes were heterogeneous among tumor nodules, suggesting a heterogeneous landscape of targetable genetic alterations among MO-HCC nodules.

We further examined several biomarkers of response to immunotherapy across unifocal HCC, TNEM and TMP. We observed elevated CD8 + T-cell exhaustion index (an indicator of dysfunctional T cells) [31] in TMP compared to TNEM and unifocal HCCs (Fig. 4b), potentially limiting the efficacy of immunotherapies in these patients. However, there was no difference in terms of TMB, immune checkpoint (PD-1/PD-L1/CTLA4) expression and intra-tumor tumor-infiltrating lymphocytes (iTILs, Supplementary Fig. 4a-b).

At the signaling level, phosphoproteomic analyses revealed consistently elevated kinase activity across tumor nodules in most IM/mixed patients (Fig. 4c). While kinase target activity was consistently high across P1 and P4 tumors, it was variable in P23, suggesting the potential of kinase inhibitory treatment for addressing inter-tumor differences in some MO-HCC patients. In most IM and MO patients, signaling pathways such as angiogenesis, MAPK signaling, and E2F targets were consistently upregulated across tumor nodules (Supplementary Fig. 4c). These convergent signaling dependencies suggest that pathway-directed therapies may provide a unifying treatment strategy, even in the presence of inter-tumoral genomic heterogeneity.

Together, these results demonstrate that integrated molecular profiling can refine patient stratification in mfHCC, identify subsets of patients with targetable alterations or shared pathway dependencies, and thereby support the implementation of precision medicine approaches in mfHCC.

### Fibrotic processes and impaired liver regeneration associated with reduced intrahepatic metastases

**Fig. 5:**
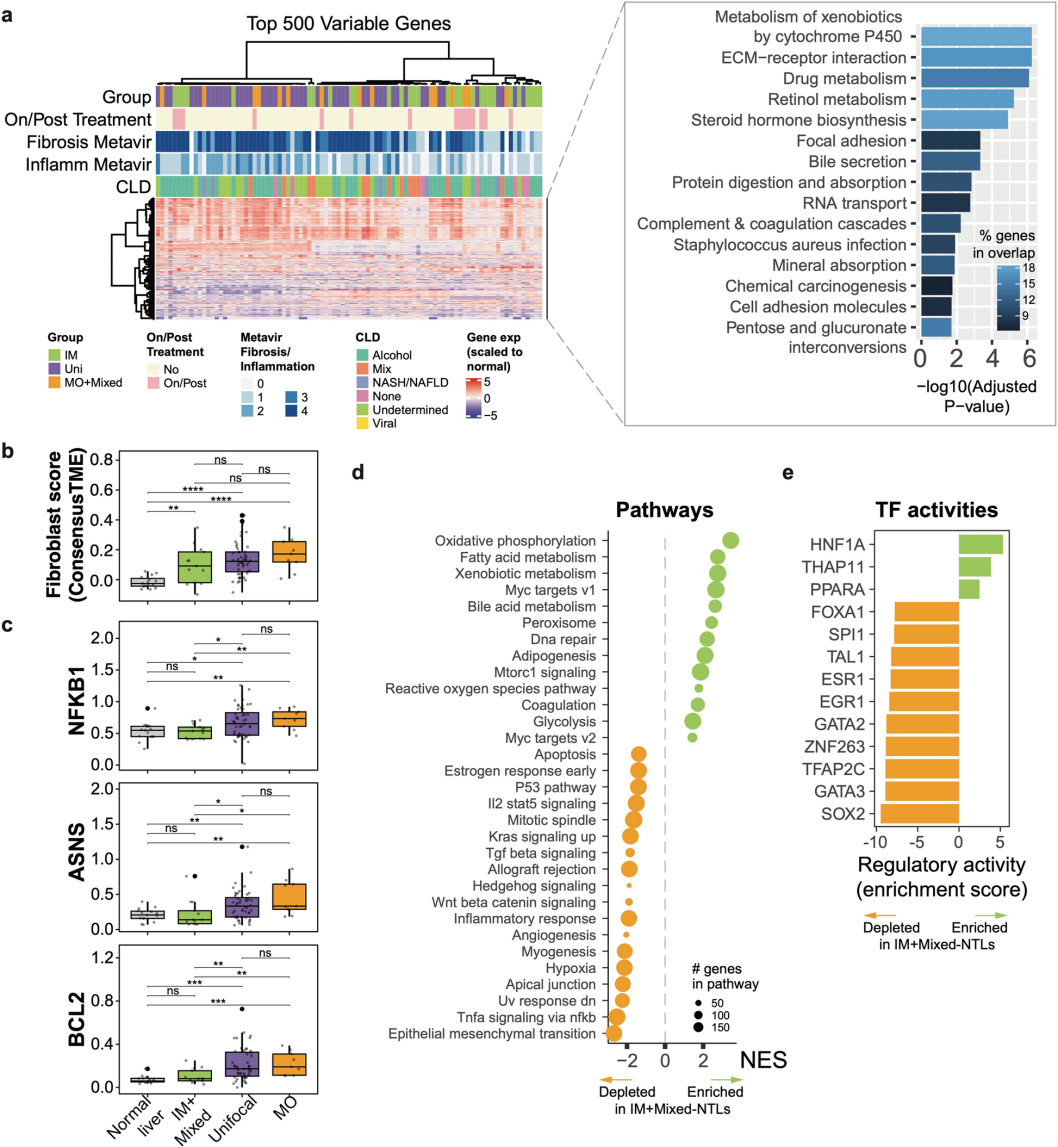
Fibrotic processes and impaired liver regeneration associated with reduced intrahepatic metastases. **a** Consensus clustering of the 500 most variable genes across the non-tumor livers (NTLs). Gene expression expressed as VST counts scaled to normal livers. The right panel depicts the 15 most enriched pathways among the 500 genes. **b**-**c** Boxplots of (**b**) fibroblast scores as defined by ConsensusTME and (**c**) gene expression of key fibroblast genes (as log2(TPM)), stratified into normal livers, IM-NTLs, unifocal-NTLs, and MO/mixed-NTLs. **d** Bubble plot of the normalized enrichment score (NES) from gene set enrichment analysis of differential expression (transcriptomic) between MO/mixed-NTLs and IM-NTLs. **e** Barplot of the regulatory activity (enrichment score) of the transcription factors enriched in MO/mixed-NTLs of IM-NTLs. **b**-**c** Statistical comparisons were performed by Mann–Whitney U tests. ****: *p* < 0.0001, ***: *p* < 0.001, **: *p* < 0.01, * *p* < 0.05, ns: not significant

In the context of mfHCC development, the liver's regenerative capacity and inflammatory milieu play pivotal roles, providing avenues for therapy or surveillance to prevent the emergence of metastases [32]. Therefore, we investigated the underlying processes within the NTL of IM-HCC (including those with mixed profiles), MO-HCC and unifocal HCC patients (hereafter IM-NTL, MO-NTL and unifocal-NTL, respectively). Enrichment analysis of the most variably expressed genes across NTL showed enrichment for ECM-receptor interactions and drug metabolism (Fig. 5a and Supplementary Fig. 5a). Among IM-NTL, MO-NTL and unifocal-NTL, IM-NTLs had the lowest fraction of fibroblasts (Fig. 5b and Supplementary Fig. 5b), consistent with their lower METAVIR fibrosis scores (Supplementary Fig. 5c). Fibro-inflammatory/immune-activated genes were similarly less enriched in IM-NTLs, as well as ECM-receptor interaction markers (e.g. *MMP9*) (Supplementary Fig. 5d-g). Notably, IM-NTLs also showed the lowest expression of markers associated with fibrogenic response (*NFKB1*) [33], damage to hepatocytes under inflammation and progression to HCC (*ASNS*) [34] and anti-apoptosis gene *BCL2* [35] (Fig. 5c). Indeed, a differential expression analysis between IM-NTLs and MO-NTLs suggests that IM-NTLs showed lower expression of genes related to myogenesis (Fig. 5d).

Moreover, IM-NTLs showed higher expression of liver-relevant metabolic pathways and DNA repair and lower expression in pathways related to epithelial-to-mesenchymal transition, TNFA via NFKB signaling, cell cycle and inflammatory response and in genes involved in liver regeneration, such as *NFKB1* and *TGFA (*Fig. 5c-d and Supplementary Fig. 5g) [36]. Accordingly, transcription factor analysis revealed lower SOX2 transcription factor activity, suggesting impaired self-renewal and increased HNF1A activity, linked to reduced regenerative capacity after inflammation [37, 38] (Fig. 5e).

Our findings showed that IM-NTLs retain more normal hepatocyte functions in a less inflamed environment than MO-NTL and unifocal-NTL, suggesting the fibrotic and inflamed environment may inhibit the intrahepatic seeding of IM.

## Discussion

In this study, we leveraged extensive multi-omics profiling of mfHCC to test whether tumors arising through IM and MO represent biologically distinct disease entities with different clinical and therapeutic implications. Previous studies have established that IM-HCC is generally more aggressive, with higher rates of vascular invasion, recurrence and poorer prognosis, whereas MO-HCC reflects independent tumor initiation within chronically diseased liver tissue. Our findings are consistent with these observations and confirm, using integrative multi-omics profiling across multiple tumor nodules, that IM-HCC and MO-HCC represent biologically distinct entities.

Beyond confirming these established clinical and pathological differences, our study provides new insights into the molecular programs that underlie these divergent behaviors. By jointly analyzing genomic, transcriptomic and (phospho)proteomic data from spatially distinct tumor nodules and matched NTL tissue, we demonstrate that metastatic capability in HCC emerges as coordinated proliferative, cell-cycle and immune-evasive programs. In parallel, we identify distinct fibrotic and regenerative states in the NTL that may differentially shape metastatic dissemination versus *de novo* tumorigenesis.

The aggressive features of IM-HCC necessitate a shift towards further systemic treatment options to address their diffuse and multifocal nature. In our cohort, we identified oncogenic and putatively targetable alterations in *PTEN*, *CDKN2A* and *ARID1A* shared across all IM lesions within patients, indicating that at least a subset of IM-HCC may be amenable to trunk-based therapeutic targeting. Moreover, IM-HCCs consistently exhibited enrichment of cell cycle-related programs, including E2F and MYC signaling, together with increased activity of mitotic regulators, in line with their higher histological grade and metastatic propensity. Preclinical studies highlight the potential of some of these as targets: TTK inhibitor CFI-402257 reduces HCC growth and metastasis by disrupting mitotic fidelity [39]; MTORC1 inhibitor sirolimus lowers recurrence post-transplant (NCT00355862) [40]. Elevated CDK1 activity within and across IM-HCC patients suggests its therapeutic potential [41], while targeting E2F signaling via CDK4/6 inhibition has demonstrated activity post-sorafenib [42]. These findings suggest that targeting aberrant cell-cycle control, potentially in combination with standard systemic therapies, could be particularly relevant in IM-HCC. Furthermore, the elevated CD8 + T-cell exhaustion observed in IM-HCC indicates that immune dysfunction may limit the efficacy of single-agent immune checkpoint blockade, providing a rationale for combination strategies that integrate immunotherapy with agents targeting proliferative or mitotic signaling. Together, these data underscore the biological basis for prioritizing systemic, combination-based therapeutic approaches in patients with IM-HCC.

Immune evasion may limit the effectiveness of single-agent anti-PD-1 therapy in HCC [31]. The elevated CD8 + T-cell exhaustion observed in IM-HCC supports combining immunotherapies with strategies that restore T-cell cytotoxicity, e.g. by targeting TTK [39]. However, the within-patient variable expression of immune checkpoint markers highlights the diverse immune responses across tumor nodules, as previously observed in multifocal HCC [43]. In fact, the authors found that smaller nodules may exhibit more active immune pathways and greater sensitivity to anti-PD-1 therapy, whereas larger nodules show enhanced proliferative signaling and resistance [43]. This further supports a dual approach incorporating anti-proliferative agents with immunotherapy for IM-HCC.

Interestingly, MO-HCC tumors within patients display a degree of molecular and histological dissimilarity comparable to tumors from different patients. Occasionally, non-clonally related tumors harbored different mutations in genes such as *CTNNB1*, but the rate of such co-occurring alterations was not higher than in tumors from different patients. Compared to IM-HCC, MO-HCC tumors show higher expression of HNF4α, a regulator of important hepatic functions [44, 45] and GATA3, which has been shown to be negatively correlated with HCC cell proliferation, migration and invasion [46]. Elevated interferon signaling has also been shown to restrain growth and invasive potential of HCC, which could help explain the lack of metastasis in MO-HCCs [47]. The lack of shared targetable genomic alterations underscores the need for multi-lesion molecular profiling to avoid under- or misclassification of targetable alterations when relying on single-biopsy approaches. On the other hand, the convergence on the dysregulation of certain kinases (e.g. CDK1) and pathways (e.g. angiogenesis) may allow distinct nodules to be treated under a unified regimen. Unlike an earlier study [17], we did not find elevated expression of inhibitory immune checkpoints in MO-HCC compared to IM-HCC, but one should acknowledge the challenges in immunophenotyping in HCC biopsies [48]. The lower T-cell exhaustion index in MO-HCC and unifocal HCC could, however, suggest that they may achieve better response to immune checkpoint inhibition.

Our study of NTLs revealed the molecular diversity in fibrosis and wound healing across mfHCC origins. The lower expression of these genes and pathways in IM-NTLs suggest that the NTL environment differs between IM-HCC and MO-HCC. Chronic inflammation and fibrosis are typically considered to promote both 'de novo' tumorigenesis in the liver and EMT, invasion, and metastasis in already established HCC [49–52]. Our findings suggest that the observed increased of pro-inflammatory cytokines, TNF-α signaling, and NF-κB activation in MO-NTLs promote hepatic stellate cell survival and ECM remodeling, thereby reinforcing fibrosis progression and connecting chronic liver injury to HCC onset [33]; this aligns with the ‘field effect’ concept, where chronic inflammation and fibrosis create a permissive tumorigenic background that supports the emergence of multiple tumors. In line with this, in DEN-induced models, in which chronic inflammation and fibrosis promote multifocal tumorigenesis, with NTLs showing elevated ECM remodeling and inflammatory signaling akin to human MO-NTLs [53]. The impaired regenerative capacity in MO-NTLs is evidenced through SOX2 activation, associated with tumor-initiating cell self-renewal, and reduced HNF1α activity, which compromises inflammation repair [38, 54]. On the contrary, we observed that the less fibrosis- and inflammation-associated and less inflamed environment of IM-NTLs may paradoxically favor metastatic dissemination, as the lack of immune cells in the tumor space may lead to reduced immune surveillance [55]. This is consistent with previous studies suggesting that the metastatic potential of IM-HCC tumors is driven by their inherent biological aggressiveness rather than being dependent on the surrounding microenvironment. Together, our results highlight that distinct fibrotic, inflammatory, and regenerative states of the non-tumor liver may differentially shape metastatic dissemination versus independent tumor initiation in HCC.

## Conclusions

This study demonstrates that mfHCC comprises biologically divergent disease entities whose behavior is determined by their evolutionary origin. IM-HCC is marked by aggressive clinical and biological behavior and T-cell exhaustion, underscoring the need for further investigations into combination therapies. Conversely, MO-HCC shows pronounced heterogeneity across nodules within a fibrotic and inflammatory liver field, but the convergence on specific kinase and pathway alterations suggests common therapeutic avenues. Importantly, IM-HCC and MO-HCC are different on the genetic, transcriptional, signaling and microenvironmental levels. Our findings provide a biological framework for understanding mfHCC heterogeneity, highlight the limitations of single-lesion sampling, and suggest that refining staging and diagnostic workflows considering multifocal origin may lead to more personalized therapeutic strategies.

## Supplementary Information

Additional file 1.

Additional file 2.

Additional file 3.

Additional file 4.

Additional file 5.

Additional file 6.

## Data Availability

Whole-exome and RNA sequencing data are available at the European Genome-phenome Archive under accessions EGAS00001005073, EGAS00001005661, EGAS00001005074 and EGAS00001005662. Proteome and phosphoproteome data are available in PRIDE (PXD025705, PXD025836 and PXD028593). Processed and normalized datasets for this study are available in zenodo (https://doi.org/10.5281/zenodo.16992981).

## Abbreviations

BCLC: Barcelona Clinic Liver Cancer
ECM: Extracellular matrix
HCC: Hepatocellular carcinoma
IM: Intrahepatic metastases
iTIL: Intra-tumor tumor-infiltrating lymphocytes
mfHCC: Multifocal HCC
MO: Multicentric occurrence
NTL: Non-tumor liver
TMB: Tumor mutational burden
TMP: Tumor with metastatic potential
TNEM: Tumor with no evidence of metastasis
