# Supplementary material for: Multi-omics profiling reveals divergent biology and liver microenvironment in HCC of metastatic and de novo origin
Source: Mol Cancer. 2026 Mar 6;25:101. doi: 10.1186/s12943-026-02604-x (PMC13078022; doi:10.1186/s12943-026-02604-x)
Supplement: Supplementary file 1 — Additional file 1. [file 12943_2026_2604_MOESM1_ESM.docx]

**Multi-omics profiling reveals divergent biology and liver microenvironments in HCC of metastatic and de novo origin**

**Gina F Boot et al**

**Supplementary Materials**

**SUPPLEMENTARY METHODS**

**Longitudinal tissue collection**

For patients identified as candidates for sorafenib treatment following tumor board discussion, a separate consent was obtained for additional study-specific biopsies. These included: (1) a pre-treatment biopsy (sometimes using the diagnostic biopsy if taken shortly before treatment initiation), (2) an on-treatment biopsy after two weeks of sorafenib, and (3) a biopsy upon tumor progression (>25% growth, indicating drug resistance). These study biopsies were not required for clinical workup. In practice, biopsy collection varied due to clinical and ethical constraints. For instance, on-treatment biopsies were not feasible if patients poorly tolerated sorafenib, requiring treatment cessation, or if patients were in poor condition, making study biopsies ethically inappropriate. Similarly, biopsies at progression were not always possible if patients deteriorated significantly. Some patients, however, consented to multiple biopsies over time, including during switches between treatments, enabling longitudinal sampling.

**Histopathological assessment**

Histopathological assessment was performed as previously described[[1]](https://paperpile.com/c/twj8g4/bvxy3). Specifically, diagnosis of HCC and histopathology evaluation were performed on FFPE slides blindly by at least two board-certified hepatopathologists (CE, MSM and/or LMT). Histopathologic grading was performed according to the Edmondson grading system[[2,3]](https://paperpile.com/c/twj8g4/9rjq2+CuGiK). Hematoxylin & eosin (H&E) slides were reviewed to define the presence or absence of cirrhosis, underlying liver disease, cholestasis, vessel infiltration, necrotic areas, major growth pattern (trabecular, solid and pseudoglandular), cytological variants and special subtypes according to the guidelines by the World Health Organization[[4]](https://paperpile.com/c/twj8g4/Blfor). Immunophenotypes (i.e. inflamed, immune-excluded and immune-desert) were classified according to Chen and Mellman[[5]](https://paperpile.com/c/twj8g4/32CwM) and as described in our previous publication[[1]](https://paperpile.com/c/twj8g4/bvxy3). Vascular growth pattern was additionally assessed in tumor tissues in terms of the Vessels Encapsulating Tumor Clusters (VETC) score (%)[[6]](https://paperpile.com/c/twj8g4/5d8i) by two histopathologists (SLR and CE). The METAVIR​ scoring system was used to assess the extent of inflammation and fibrosis[[7]](https://paperpile.com/c/twj8g4/LQ0Y).

**DNA and RNA extraction**

DNA and RNA extraction, quantification and RNA integrity assessment were performed as in our previous publication[[1]](https://paperpile.com/c/twj8g4/bvxy3). Specifically, genomic DNA and total RNA from tumor and adjacent liver parenchyma were extracted using the ZR-Duet DNA and RNA MiniPrep Plus kit (Zymo Research); total RNA of 15 liver biopsies with normal histology was extracted using Trizol (Thermo Fisher Scientific). Extracted DNA was quantified using the Qubit Fluorometer (Invitrogen). Extracted RNA was quantified using NanoDrop 2000 spectrophotometer (Thermo Fisher Scientific), and RNA quality/integrity was assessed with an Agilent 2100 BioAnalyzer using RNA 6000 Nano Kit (Agilent Technologies).

**Whole-exome sequencing and data processing**

All 76 HCC biopsies and 44 non-tumoral tissues from 22 patients were subjected to whole-exome sequencing, as previously described[[1]](https://paperpile.com/c/twj8g4/bvxy3). Specifically, whole-exome capture was performed using the SureSelectXT Clinical Research Exome (Agilent Technologies) or SureSelect Human All Exon V6+COSMIC (Agilent Technologies) platforms and paired-end 101-bp sequencing was performed on an Illumina HiSeq 2500 at the Genomics Facility Basel. Tumor biopsies and non-tumoral biopsies were sequenced to median depths of 118 (range 16.4-267) and 53.3 (range 34.5-132) inclusive of the unifocal HCC cohort, respectively.

Sequencing data analysis was performed as in our previous study[[1]](https://paperpile.com/c/twj8g4/bvxy3). Sequence reads were aligned to the reference human genome GRCh37 using Burrows-Wheeler Aligner (BWA, v0.7.12/13)[[8]](https://paperpile.com/c/twj8g4/8bT6K). Local realignment, duplicate removal and base quality adjustment were performed using the Genome Analysis Toolkit (GATK, v3.6)[[9]](https://paperpile.com/c/twj8g4/wDUsD) and Picard (<http://broadinstitute.github.io/picard/>, v2.4.1). Somatic single nucleotide variants (SNVs) and small insertions and deletions (indels) were detected using MuTect (v1.1.4)[[10]](https://paperpile.com/c/twj8g4/jfHkR) and Strelka (v1.0.15)[[11]](https://paperpile.com/c/twj8g4/TA75K), respectively. We filtered single nucleotide variants (SNVs) and indels from the WES data based on variant allelic fraction (VAF) and read depth. Specifically, variants with a tumor VAF <5% and/or supported by fewer than 3 reads were excluded from further analysis. Additionally, we filtered out variants for which the tumor VAF was less than 5 times that of the paired non-tumor sample. We further excluded variants identified in at least two of a panel of 123 non-tumor samples, including the 115 non-tumor samples included in the current study, captured and sequenced using the same protocols using the artefact detection mode of MuTect2 implemented in GATK 3.6. We annotated mutations if they are in mutational driver gene according to Schulze et al[[12]](https://paperpile.com/c/twj8g4/54Un), Fujimoto et al[[13]](https://paperpile.com/c/twj8g4/KGvE), and the HCC subsets of Martincorena et al[[14]](https://paperpile.com/c/twj8g4/4aOq), Bailey et al [[15]](https://paperpile.com/c/twj8g4/FmQa), and Martinez-Jimenez et al [[16]](https://paperpile.com/c/twj8g4/8tEa).

Allele-specific CNAs were identified using FACETS (v0.5.5)[[17]](https://paperpile.com/c/twj8g4/Vlpmm), which performs a joint segmentation of the total and allelic copy ratio and infers allele-specific copy number states. Copy number states were collapsed to the gene level based on the median values to coding gene resolution based on all coding genes retrieved from the Ensembl (release GRCh37.p13). Genes with total copy number greater than gene-level median ploidy were considered gains; greater than ploidy + 4, amplifications; less than ploidy, losses; and total copy number of 0, homozygous deletions. Tumors with >5% of the genome at copy number 0 (homozygous deletions, 5 tumors) were excluded from the identification of homozygous deletions and from the computation of fraction of genome altered. Fraction of genome altered was computed as the fraction of genes with amplification, gain, loss or deletion.

**RNA-sequencing and data processing**

All 76 HCC tumor biopsies and 44 non-tumoral tissues from 22 HCC patients and 15 normal livers were subjected to RNA-sequencing, as previously described[[1]](https://paperpile.com/c/twj8g4/bvxy3). RNA-seq library prep was performed using the TruSeq Stranded Total RNA Library Prep Kit with Ribo-Zero Gold (Illumina). Single-end 126-bp sequencing was performed on an Illumina HiSeq 2500 using v4 SBS chemistry at the Genomics Facility Basel.

Sequencing data analysis was performed as in our previous study[[1]](https://paperpile.com/c/twj8g4/bvxy3). Sequence reads were aligned simultaneously to the human reference genome GRCh37, HBV strain ayw genome (NC 003977.2), and HCV genotype 1 genome (NC 004102.1) by STAR (v2.5.2a)[[18]](https://paperpile.com/c/twj8g4/7cu6h) using the two-pass approach. Median numbers of reads aligning to the human genome were 53.6 million (range 42.3 - 124 million), 55.2 million (range 37.5 - 82.5 million) and 63.5 million (range 52.5- 82.2 million) for the HCC and non-tumor inclusive of the unifocal HCC cohort, and normal liver biopsies, respectively (**Supplementary Table 1**). Transcript quantification was performed using RSEM (v1.2.31)[[19]](https://paperpile.com/c/twj8g4/BTyoR). Molecular subtyping according to Hoshida *et al*[*[20]*](https://paperpile.com/c/twj8g4/8ZkQL) was performed using the Nearest Template Prediction (<http://software.broadinstitute.org/cancer/software/genepattern>) [[20]](https://paperpile.com/c/twj8g4/8ZkQL). Raw RSEM-expected counts were adjusted for differences in sequencing depth by applying the DESeq2 median-of-ratios method and were further transformed by variance stabilizing transformation into log2 space. The logTPM counts (transcripts per million) were calculated from the DESEq2-normalised counts using gene lengths. These were used to compare individual gene expressions across sample groups.

**Proteomic and Phosphoproteomic data processing**

Log2-transformed, median subtracted proteomics and phosphoproteomics data were collected from Ng et al[[1]](https://paperpile.com/c/twj8g4/bvxy3) and Dazert et al[[21]](https://paperpile.com/c/twj8g4/4CCy). We retrieved 22 HCCs and 16 non-tumor livers from 9 mfHCC patients (6 from Ng et al[[1]](https://paperpile.com/c/twj8g4/bvxy3) and 3 from Dazert et al[[21]](https://paperpile.com/c/twj8g4/4CCy)). As comparison groups, proteomic and phosphoproteomic data were obtained for 22 HCCs and 22 non-tumor livers from unifocal patients and 5 normal liver samples, all from Ng et al[[1]](https://paperpile.com/c/twj8g4/bvxy3). Two unifocal patients did not have phosphoproteomic data.

The proteomics and phosphoproteomics data for the two studies were measured by different proteomic platforms. For each study, proteins were retained if they were detected in at least 50% of the samples. Proteins meeting this criterion in both studies were included, and the final filtered dataset contained 5,194 proteins. To adjust for batch effects across the two studies, the *removeBatchEffect* function from the *limma* package (v3.52.4)[[22]](https://paperpile.com/c/twj8g4/EJG1) was applied to the filtered data matrix. Missing values were imputed in the batch-corrected dataset by sampling from a left-shifted Gaussian distribution. For each protein, missing values were replaced with random numbers drawn from a normal distribution with a mean downshifted by 1.8 standard deviations from the sample mean and a standard deviation scaled to 30% of the sample's standard deviation. This approach simulates low-intensity signals while accounting for the missing-not-at-random (MNAR) nature of the data [[23]](https://paperpile.com/c/twj8g4/0MGh).

Filtering, batch correction, missing data imputation were performed for the phosphosite data in the same manner as described for proteins. To normalize phosphosites for overall protein levels, we computed the difference between the phosphorylation site levels and protein levels, for 1957 phosphosites where the proteins were detected by both technologies.

**Analysis of clonal relationship**

The BreakClone method[[24]](https://paperpile.com/c/twj8g4/Jw0km) was used to assess the clonal relationships between tumor pairs. BreakClone calculates the probability of two tumor samples being clonally related by analyzing the allele frequencies of shared and private mutations between the samples. To account for the influence of variant population frequency, each mutation was weighted, with lower population frequency variants given higher predictive value. We adjusted these weights using both mutation calls from The Cancer Genome Atlas (TCGA) liver cancer cohort and mutations identified within our own cohort. The resulting concordance score, indicating clonal relatedness, ranges from 0 (no genomic similarity) to 1 (highly similar mutation profiles). A reference distribution of concordance scores, calculated from 2,680 tumor pairs from different patients (inter-patient tumor pairs; range 0–0.05), was used for permutation testing to determine the significance of each tumor pair's score. Tumor pairs were classified as clonally related (P < 0.01), ambiguous (0.01 < P < 0.05), or non-clonal (P > 0.05). To provide a comparison for the clonal and non-clonal intra-patient tumor pairs, we randomly sampled 50 tumor pairs from the set of inter-patient tumor pairs, though note that not all clinicopathological parameters were available for all inter-patient pairs.

## **Tumor mutational burden (TMB), mutational signatures and actionable alterations**

TMB was defined as the number of somatic non-synonymous mutations per megabase (MB) of sequenced exonic regions. Fraction of genome altered was computed as the fraction of genes with amplification, gain, loss or deletion. We then used SigProfilerSingleSample (v0.0.0.27)[[25]](https://paperpile.com/c/twj8g4/S6v0C) on counts of tri-nucleotide contexts for samples with more than 50 mutations to identify contributions of known COSMIC mutational signatures (v3.3.1) across tumor samples. We annotated the alterations (somatic mutations, CNAs and TMB) identified in our cohort with OncoKB[[26]](https://paperpile.com/c/twj8g4/AUYme) to highlight potentially actionable alterations and prioritise variants that are linked to investigational drugs in HCC and other solid malignancies, with therapeutic level of evidence 4 (predictive of drug response based on preliminary data).

**Differential expression, pathway and transcription factor activity analyses**

Differential expression analysis was conducted on raw RSEM-expected counts using DESeq2 (v1.36.0)[[28]](https://paperpile.com/c/twj8g4/myoQx). To filter out low-abundance genes, only genes with a minimum count of 10 in at least 70% of the samples from the smallest group were retained for analysis. Differential expression analysis was then performed comparing TNEM vs. TMP, or MO+Mixed vs IM-NTLs, accounting for treatment status (pre-treatment vs. on/post-treatment). The Wald test was used to calculate p-values, and results were filtered at a significance threshold of adjusted p-value (FDR) < 0.05. The t-statistic generated by DESeq2 was used as input for GSEA for MSigDB hallmark gene sets from msigdbr (v7.5.1) using the R package fgsea (v1.24.0)[[29]](https://paperpile.com/c/twj8g4/7nV6b) with default parameters. Gene sets with FDR < 0.05 were considered statistically significantly enriched. The t-statistic from differential expression analysis was also used as input to estimate the differential transcription factor (TF) activities between groups, using the decoupleR package (v2.2.2)[[30]](https://paperpile.com/c/twj8g4/LVcl9) to infer TF activities based on the WMEAN method, using weighted contributions of target genes in the differential expression dataset. Experimentally validated TF-target regulatory networks were extracted from the Dorothea database with confidence levels A-C [[31]](https://paperpile.com/c/twj8g4/93JcO). The resultant activity scores were normalized, and statistical significance (permutation-derived p-value) was assessed through 1000 random permutations with p <0.05 considered as significant.

Single-sample Gene Set Enrichment Analysis was performed on variance-stabilized transformed (VST) RNA-seq counts or log2 normalized proteomics data to infer pathway activity for each sample, using the GSVA package (v1.44.5). The pathway gene sets were obtained from the MSigDB database [[32,33]](https://paperpile.com/c/twj8g4/e1s1+PKrn), using only gene sets with at least 10 genes. Pathway scores were then scaled to the scores of normal tissue, and averaged per TMP/TNEM or non-tumor liver.

**Analysis of the tumor microenvironment**

TPM counts were input into the ConsensusTME (version 0.0.1.9000)[[34]](https://paperpile.com/c/twj8g4/Aw52F) tool to estimate the relative abundance of various cell types within the tumor microenvironment, using the TCGA LIHC (Liver Hepatocellular Carcinoma) dataset as the cancer-specific signature matrix. Analysis with the ESTIMATE, xCell and consensusTME enrichment tool was performed[[34–36]](https://paperpile.com/c/twj8g4/3vOhb+Aw52F+vrW2), as well as gsva (single sample gene set analysis, ‘ssgsea’, method) analysis with inflammatory markers of the liver [[37,38]](https://paperpile.com/c/twj8g4/eaSlg+lPgfp). The resulting cell-type enrichment scores represent the relative levels of immune and stromal populations across samples. We quantified CD8+ T-cell exhaustion using a previously published T-cell exhaustion index (TEI) model, constructed using the expression of *EEF1E1*, *GAGE1*, *CHORDC1*, *IKBIP*, and *MAGOH* [*[39]*](https://paperpile.com/c/twj8g4/MUW6V). We calculated the TEI for each sample in our bulk RNA-seq dataset using TPM (transcripts per million)-normalized gene expression counts, determined as a weighted sum of the expression levels of the five genes, applying the coefficients provided by the original study.

**Kinase-Substrate Enrichment Analysis (KSEA)**

KSEA[[40]](https://paperpile.com/c/twj8g4/xTvSp) was performed on the normalized phosphosite data using the KSEAapp R package (v0.99.0)[[41]](https://paperpile.com/c/twj8g4/n45GM). The analysis utilized an updated Kinase-Substrate dataset combining the PhosphoSitePlus (2022) and NetworKIN (2016) databases, limited to human kinase-substrate relationships. For each sample, kinase activity Z-scores were calculated with the NetworKIN score cutoff set to 5.

**Statistical Analysis**

Principal component analysis (PCA) of the first two components was conducted on the 500 most variable genes, proteins, or phosphosites using the prcomp function from the base stats package. Euclidean distances between samples or to the median of normal samples were calculated using the dist function from the base stats package. Spearman's correlation was used to assess relationships between continuous variables. The ConsensusClusterPlus [[42]](https://paperpile.com/c/twj8g4/731Ab) package (v1.60.0) and silhouette method was used for consensus clustering on the 500 most variable genes based on their standard deviation, on vst-transformed counts of both tumors and NTLs scaled to normal livers. KEGG 2021 pathway enrichment analysis was performed on the most variable genes using the rba_enrichr function from the *rbioapi* R package (v0.8.1) [[43]](https://paperpile.com/c/twj8g4/2JlGs).

Comparisons of ordinal clinicopathological variables (e.g. BCLC or Edmondson grade) were performed using Mann–Whitney U tests. Comparisons of categorical variables (including frequency of copy number gains and losses) were performed using Fisher’s exact tests or chi-squared tests. Comparisons of numerical variables were performed using Mann–Whitney U test. To compare numerical variables across at least three groups, the Kruskal-Wallis test was first applied to assess differences in distribution among the groups. Post-hoc pairwise comparisons between the groups were conducted using the pairwise Wilcoxon rank-sum test with a Benjamini-Hochberg correction to control the FDR, using the stats package. Overall survival (OS) was defined as the time (in days) from diagnosis to death (or last follow-up, for censored observations). OS analysis was conducted using Kaplan-Meier survival curves using the survfit function from the survival package (v3.5.7). Comparisons of OS were performed using the log-rank test and visualized using the ggsurvplot function from the survminer package (v0.4.9). All statistical tests were two-sided unless otherwise indicated, and p ≤ 0.05 was considered statistically significant.

**SUPPLEMENTARY FIGURES**

**
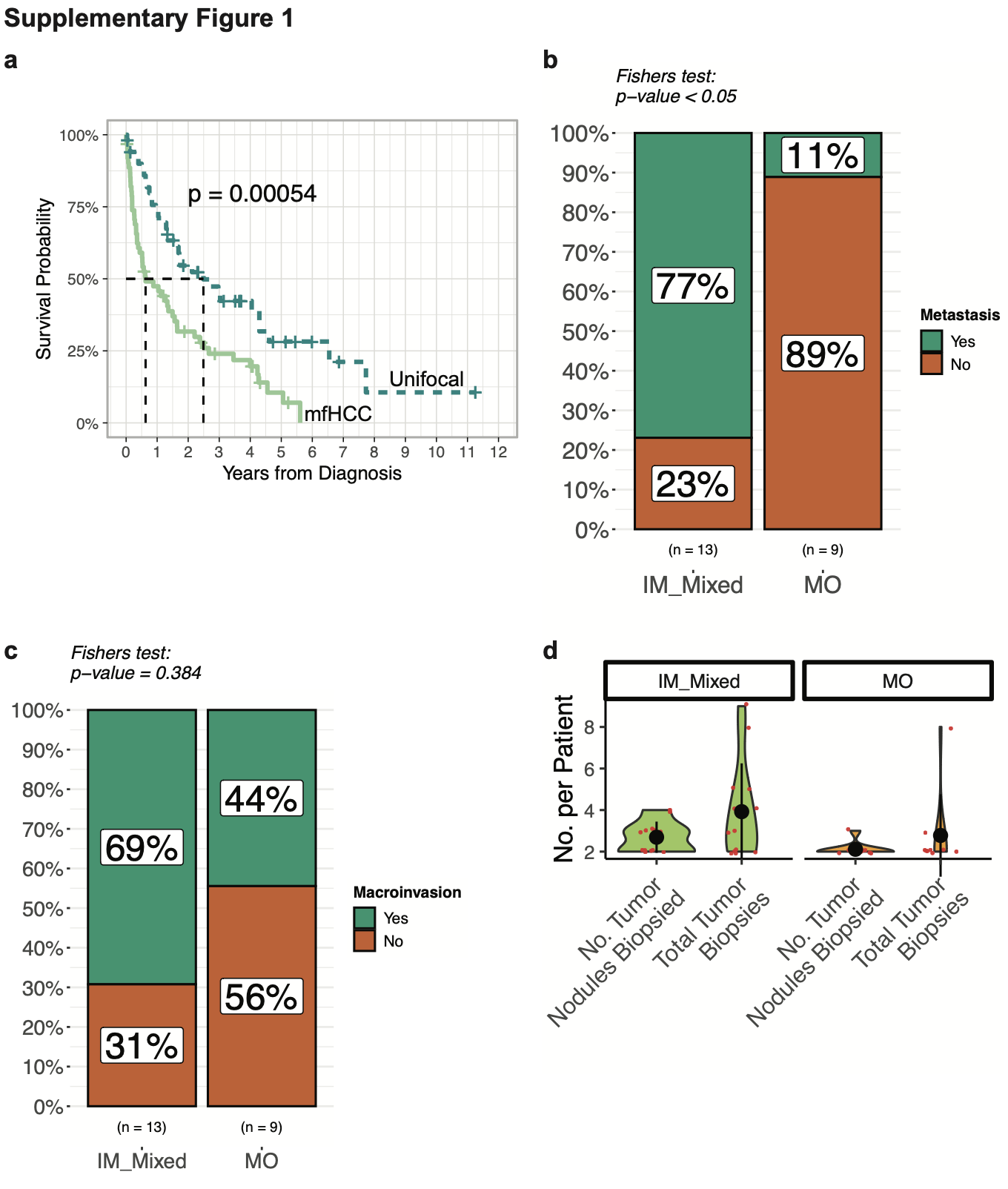
**

**Supplementary Figure 1: a)** Kaplan-Meier curves and log-rank p value, showing overall survival (days from diagnosis) analysis between multifocal HCC and unifocal HCC patients. **b-c)** Stacked bar plots illustrating the percentage of patients with **(b)** extrahepatic metastasis and **(c)** macrovascular invasion, at diagnosis or during the follow-up period, stratified into patients with IM or mixed profiles, patients with MO profile and patients with unifocal HCC. **d)** The number of unique tumor nodules identified via imaging or confirmed by pathological examination, compared to the number of biopsies obtained, per patient. Statistical comparisons were performed by **(a)** log-rank test, **(b-c)** Fisher's exact tests and **(d)** Mann-Whitney U tests.


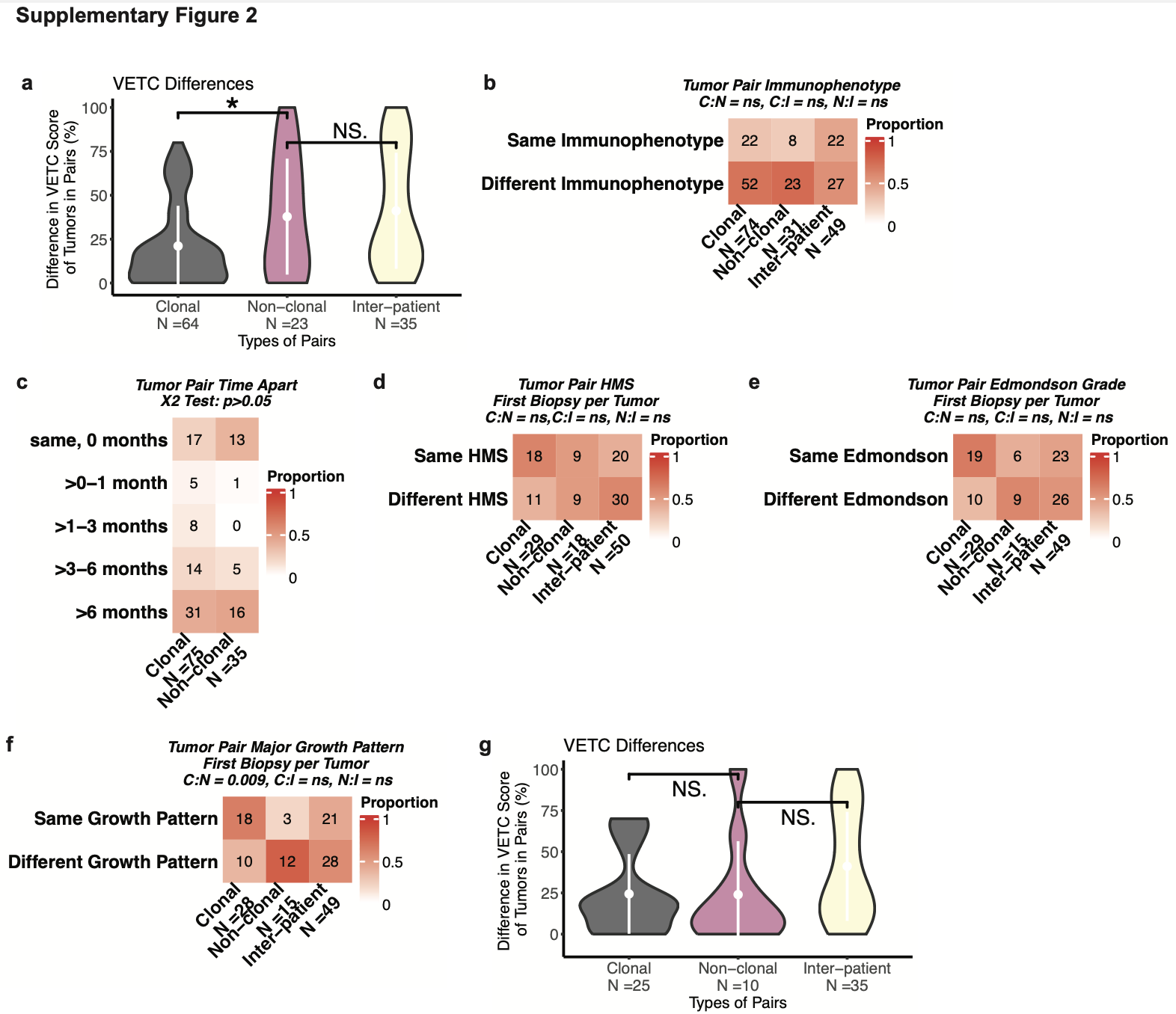


**Supplementary Figure 2: a)** Violin plot illustrating the pairwise differences between the VETC scores of clonal, non-clonal, and random inter-patient biopsy pairs. **b)** Heatmap of the pairwise comparisons of immunophenotypes between clonal, non-clonal and random inter-patient biopsy pairs. **c)** Heatmap of the pairwise comparisons of the length of time between biopsies between clonal and non-clonal biopsy pairs. **d-f)** Heatmaps of the pairwise comparisons of **(d)** Hoshida molecular subtypes, **(e)** Edmondson grade and **(f)** histological growth pattern between biopsies between clonal, non-clonal biopsy pairs. Statistical comparisons were performed by **(a,g)** Mann-Whitney U tests, **(b,d-f)** Fisher's exact tests and **(c)** chi-squared test.


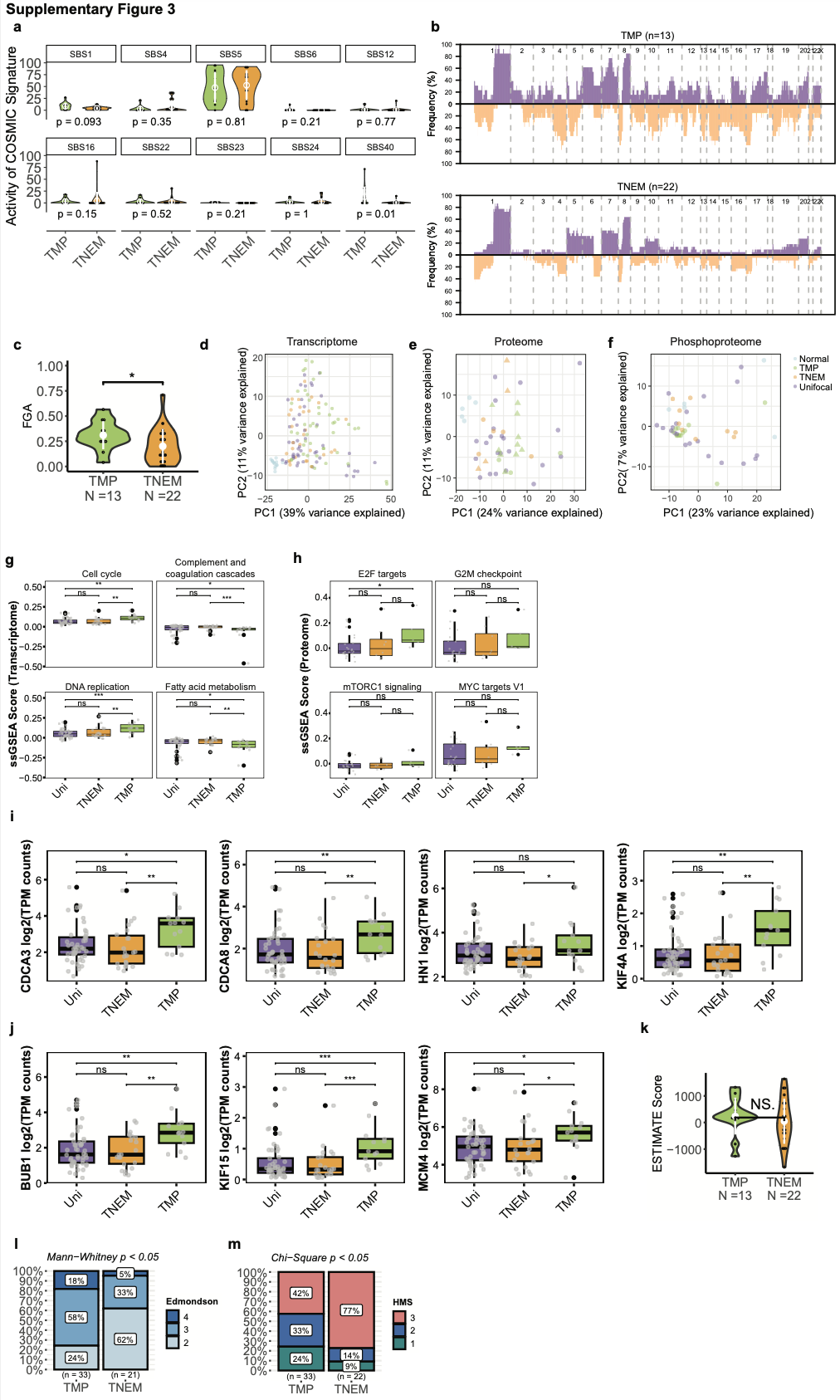


**Supplementary Figure 3: a)** Comparisons of mutational signature activities for signatures previously implicated in HCC carcinogenesis (activities for biopsy samples averaged for each TMP/TNEMs). **b)** Comparison of frequencies of copy number gains (purple bars) and losses (orange bars) across the genome (the maximum absolute copy number states of the biopsies were used per TMP/TNEM). **c)** Comparison of the fraction of genome altered by copy number alterations (FGA, the average FGA was used per TMP/TNEM). **d-f)** Principal component analyses of the 500 most variable features of the (**d**) transcriptome, (**e**) proteome and (**f**) phosphoproteome, showing the first two principal components. Normal livers, TMP, TNEM and unifocal HCCs labelled as in color key. **g-h)** Boxplots of the single-sample gene set enrichment analysis (ssGSEA) scores of the (**g**) transcriptome and (**h**) proteome for select pathways. **i-j)** Expression of (**i**) signature genes of E2F targets[[44]](https://paperpile.com/c/twj8g4/gROU7), and (**j**) cell cycle-related genes[[45–48]](https://paperpile.com/c/twj8g4/inJ5z+zHv7s+Lf7yo+l8rVA), stratified into TMP, TNEM and unifocal HCC. **k)** ESTIMATE score per TMP/TNEM. **l-m)** Comparisons of the histological (Edmondson) grades and Hoshida molecular subtypes between biopsies from individual nodules of TMP and biopsies from TNEM. For each tumor nodule, the first (i.e. at the first timepoint) biopsy was used for the comparisons. Statistical comparisons were performed by **(a, g-l**) Mann-Whitney U tests and **(m**) Chi-squared test.


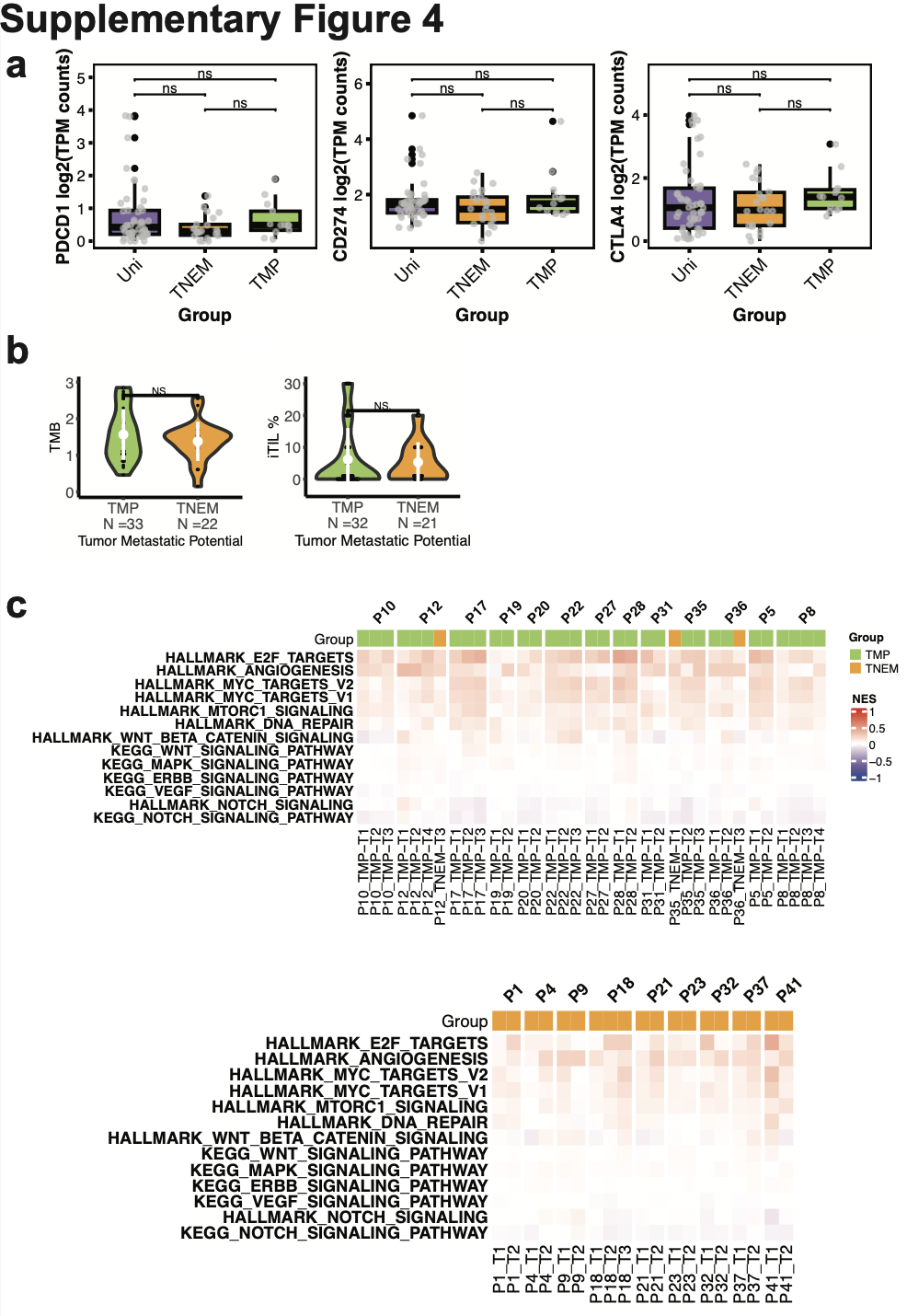


**Supplementary Figure 4: a)** Gene expression of PDCD1 (PD-1), CD274 (PD-L1) and CTLA4 as log2(TPM counts), stratified into TMP, TNEM and unifocal HCC, **b)** Violin plots of tumor mutation burden (TMB, left) and intratumoral infiltrating lymphocytes (iTIL, right) in TMPs and TNEMs. **c)** Heatmaps of normalised enrichment score (NES) of KEGG pathways from single sample gene set enrichment (transcriptomic) scaled to normal liver in TMPs (in IM and Mixed patients, top) and TNEMs (MO patients, bottom). Statistical comparisons in **(a,b)** were performed by Mann-Whitney U tests.


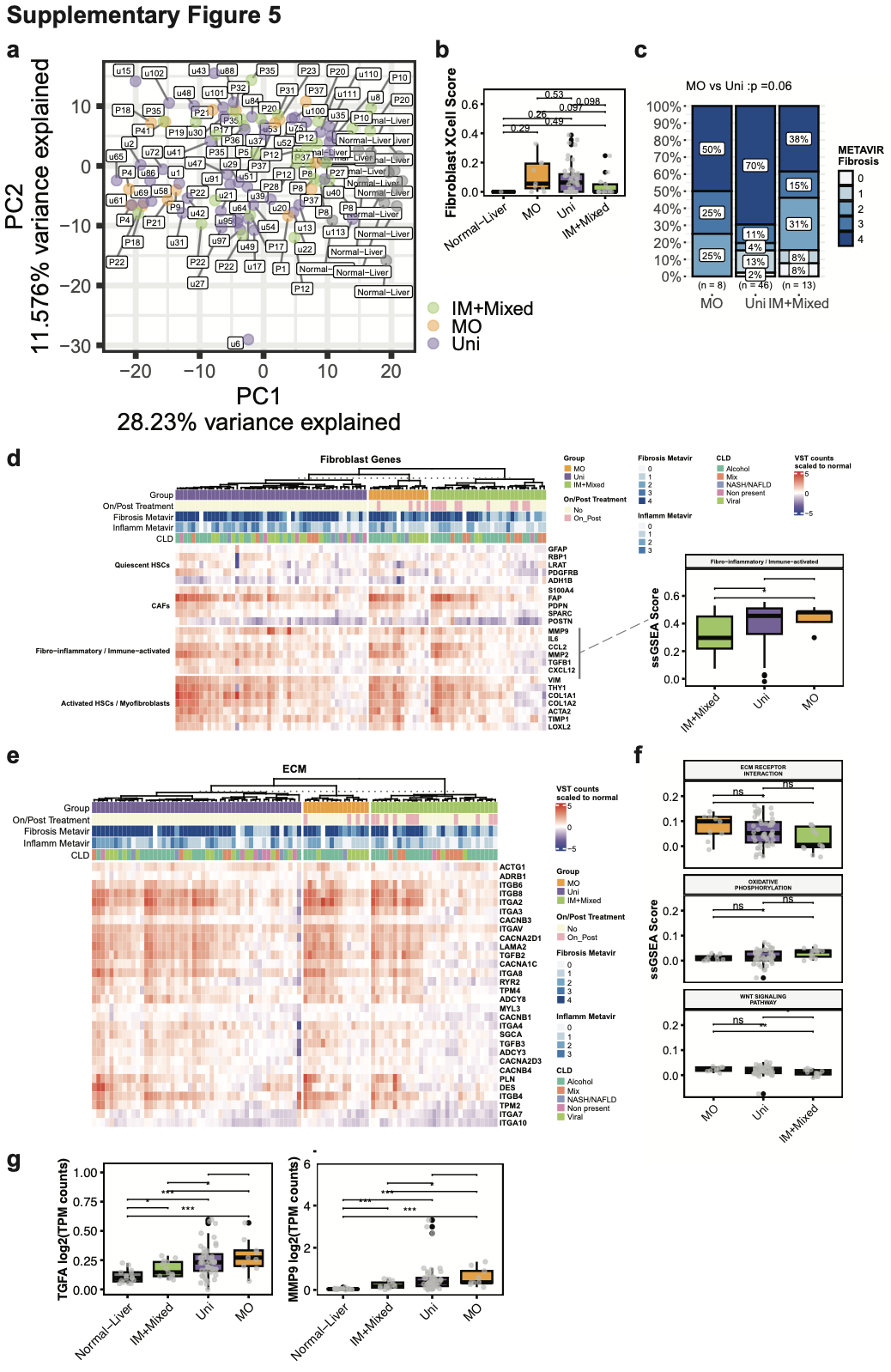


**Supplementary Figure 5: a)** Principal component analyses of the 500 most variable features of the transcriptome, showing the first two principal components. Normal livers, and NTLs from IM+Mixed patients, MO patients and unifocal patients, labelled as in color key. **b)** Boxplot of fibroblast scores as defined by xCell, stratified into normal livers, MO-NTLs, unifocal-NTLs, andIM/mixed-NTLs. **c)** Stacked bar plots illustrating the percentage of patients with each METAVIR Fibrosis score (at diagnosis), stratified into patients with IM or mixed profiles, patients with MO profile and patients with unifocal HCC. **d)** Heatmap of the expression of fibroblast-associated genes, stratified into IM+Mixed, MO and unifocal NTLs, with clinico-pathological features annotated as per the color key. Boxplot (right of heatmap) of the single-sample gene set enrichment analysis (ssGSEA) scores of the genes labelled as Fibro-inflammatory/Immune-activated. **e)** Heatmap of the expression of leading-edge genes of the ECM-receptor interaction pathway, stratified into IM+Mixed, MO and unifocal NTLs, with clinico-pathological features annotated as per the color key. **f)** Boxplots of the single-sample gene set enrichment analysis (ssGSEA) scores of the transcriptome, for select pathways. **g)** Boxplots of gene expression of genes less abundant in IM-NTL, stratified into normal livers, MO-NTLs, unifocal-NTLs, and IM/Mixed-NTLs. Statistical comparisons were performed by **(b-d,f-g)** Mann-Whitney U tests.

**REFERENCES**

[1. Ng CKY, Dazert E, Boldanova T, Coto-Llerena M, Nuciforo S, Ercan C, et al. Integrative proteogenomic characterization of hepatocellular carcinoma across etiologies and stages. Nat Commun. 2022;13:2436.](http://paperpile.com/b/twj8g4/bvxy3)

[2. Nuciforo S, Fofana I, Matter MS, Blumer T, Calabrese D, Boldanova T, et al. Organoid Models of Human Liver Cancers Derived from Tumor Needle Biopsies. Cell Rep. 2018;24:1363–76.](http://paperpile.com/b/twj8g4/9rjq2)

[3. Edmondson HA, Steiner PE. Primary carcinoma of the liver: a study of 100 cases among 48,900 necropsies. Cancer. 1954;7:462–503.](http://paperpile.com/b/twj8g4/CuGiK)

[4. World Health Organization. WHO Classification of Tumours of the Digestive System. World Health Organization; 2010.](http://paperpile.com/b/twj8g4/Blfor)

[5. Chen DS, Mellman I. Elements of cancer immunity and the cancer-immune set point. Nature. 2017;541:321–30.](http://paperpile.com/b/twj8g4/32CwM)

[6. Renne SL, Woo HY, Allegra S, Rudini N, Yano H, Donadon M, et al. Vessels encapsulating tumor clusters (VETC) is a powerful predictor of aggressive hepatocellular carcinoma. Hepatology. 2020;71:183–95.](http://paperpile.com/b/twj8g4/5d8i)

[7. Bedossa P, Poynard T. An algorithm for the grading of activity in chronic hepatitis C. The METAVIR Cooperative Study Group. Hepatology. 1996;24:289–93.](http://paperpile.com/b/twj8g4/LQ0Y)

[8. Li H, Durbin R. Fast and accurate short read alignment with Burrows-Wheeler transform. Bioinformatics. 2009;25:1754–60.](http://paperpile.com/b/twj8g4/8bT6K)

[9. McKenna A, Hanna M, Banks E, Sivachenko A, Cibulskis K, Kernytsky A, et al. The Genome Analysis Toolkit: a MapReduce framework for analyzing next-generation DNA sequencing data. Genome Res. 2010;20:1297–303.](http://paperpile.com/b/twj8g4/wDUsD)

[10. Cibulskis K, Lawrence MS, Carter SL, Sivachenko A, Jaffe D, Sougnez C, et al. Sensitive detection of somatic point mutations in impure and heterogeneous cancer samples. Nat Biotechnol. 2013;31:213–9.](http://paperpile.com/b/twj8g4/jfHkR)

[11. Saunders CT, Wong WSW, Swamy S, Becq J, Murray LJ, Cheetham RK. Strelka: accurate somatic small-variant calling from sequenced tumor-normal sample pairs. Bioinformatics. 2012;28:1811–7.](http://paperpile.com/b/twj8g4/TA75K)

[12. Schulze K, Imbeaud S, Letouzé E, Alexandrov LB, Calderaro J, Rebouissou S, et al. Exome sequencing of hepatocellular carcinomas identifies new mutational signatures and potential therapeutic targets. Nat Genet. 2015;47:505–11.](http://paperpile.com/b/twj8g4/54Un)

[13. Fujimoto A, Furuta M, Totoki Y, Tsunoda T, Kato M, Shiraishi Y, et al. Whole-genome mutational landscape and characterization of noncoding and structural mutations in liver cancer. Nat Genet. 2016;48:500–9.](http://paperpile.com/b/twj8g4/KGvE)

[14. Martincorena I, Raine KM, Gerstung M, Dawson KJ, Haase K, Van Loo P, et al. Universal Patterns of Selection in Cancer and Somatic Tissues. Cell. 2017;171:1029–41.e21.](http://paperpile.com/b/twj8g4/4aOq)

[15. Bailey MH, Tokheim C, Porta-Pardo E, Sengupta S, Bertrand D, Weerasinghe A, et al. Comprehensive Characterization of Cancer Driver Genes and Mutations. Cell. 2018;174:1034–5.](http://paperpile.com/b/twj8g4/FmQa)

[16. Martínez-Jiménez F, Muiños F, Sentís I, Deu-Pons J, Reyes-Salazar I, Arnedo-Pac C, et al. A compendium of mutational cancer driver genes. Nat Rev Cancer. 2020;20:555–72.](http://paperpile.com/b/twj8g4/8tEa)

[17. Shen R, Seshan VE. FACETS: allele-specific copy number and clonal heterogeneity analysis tool for high-throughput DNA sequencing. Nucleic Acids Res. 2016;44:e131.](http://paperpile.com/b/twj8g4/Vlpmm)

[18. Dobin A, Davis CA, Schlesinger F, Drenkow J, Zaleski C, Jha S, et al. STAR: ultrafast universal RNA-seq aligner. Bioinformatics. 2013;29:15–21.](http://paperpile.com/b/twj8g4/7cu6h)

[19. Li B, Dewey CN. RSEM: accurate transcript quantification from RNA-Seq data with or without a reference genome. BMC Bioinformatics. 2011;12:323.](http://paperpile.com/b/twj8g4/BTyoR)

[20. Hoshida Y, Nijman SMB, Kobayashi M, Chan JA, Brunet J-P, Chiang DY, et al. Integrative transcriptome analysis reveals common molecular subclasses of human hepatocellular carcinoma. Cancer Res. 2009;69:7385–92.](http://paperpile.com/b/twj8g4/8ZkQL)

[21. Dazert E, Boldanova T, Ng CKY, Colombi M, Wieland S, Rosenberger G, et al. Multi-omics of sorafenib responsiveness in HCC patients [Internet]. medRxiv. 2025. Available from:](http://paperpile.com/b/twj8g4/4CCy) <http://medrxiv.org/lookup/doi/10.1101/2025.07.15.25331324>

[22. Ritchie ME, Phipson B, Wu D, Hu Y, Law CW, Shi W, et al. limma powers differential expression analyses for RNA-sequencing and microarray studies. Nucleic Acids Res. 2015;43:e47.](http://paperpile.com/b/twj8g4/EJG1)

[23. Tyanova S, Temu T, Sinitcyn P, Carlson A, Hein MY, Geiger T, et al. The Perseus computational platform for comprehensive analysis of (prote)omics data. Nat Methods. 2016;13:731–40.](http://paperpile.com/b/twj8g4/0MGh)

[24. Lips EH, Kumar T, Megalios A, Visser LL, Sheinman M, Fortunato A, et al. Genomic analysis defines clonal relationships of ductal carcinoma in situ and recurrent invasive breast cancer. Nat Genet. 2022;54:850–60.](http://paperpile.com/b/twj8g4/Jw0km)

[25. Díaz-Gay M, Vangara R, Barnes M, Wang X, Islam SMA, Vermes I, et al. Assigning mutational signatures to individual samples and individual somatic mutations with SigProfilerAssignment. Bioinformatics [Internet]. 2023;39. Available from:](http://paperpile.com/b/twj8g4/S6v0C) <http://dx.doi.org/10.1093/bioinformatics/btad756>

[26. Chakravarty D, Gao J, Phillips SM, Kundra R, Zhang H, Wang J, et al. OncoKB: A Precision Oncology Knowledge Base. JCO Precis Oncol [Internet]. 2017;2017. Available from:](http://paperpile.com/b/twj8g4/AUYme) <http://dx.doi.org/10.1200/PO.17.00011>

[27. Wintersinger JA, Dobson SM, Kulman E, Stein LD, Dick JE, Morris Q. Reconstructing Complex Cancer Evolutionary Histories from Multiple Bulk DNA Samples Using Pairtree. Blood Cancer Discov. 2022;3:208–19.](http://paperpile.com/b/twj8g4/PxBS)

[28. Love MI, Huber W, Anders S. Moderated estimation of fold change and dispersion for RNA-seq data with DESeq2. Genome Biol. 2014;15:550.](http://paperpile.com/b/twj8g4/myoQx)

[29. Korotkevich G, Sukhov V, Budin N, Shpak B, Artyomov MN, Sergushichev A. Fast gene set enrichment analysis [Internet]. bioRxiv. bioRxiv; 2016. Available from:](http://paperpile.com/b/twj8g4/7nV6b) <http://biorxiv.org/lookup/doi/10.1101/060012>

[30. Badia-I-Mompel P, Vélez Santiago J, Braunger J, Geiss C, Dimitrov D, Müller-Dott S, et al. decoupleR: ensemble of computational methods to infer biological activities from omics data. Bioinform Adv. 2022;2:vbac016.](http://paperpile.com/b/twj8g4/LVcl9)

[31. Garcia-Alonso L, Holland CH, Ibrahim MM, Turei D, Saez-Rodriguez J. Benchmark and integration of resources for the estimation of human transcription factor activities. Genome Res. 2019;29:1363–75.](http://paperpile.com/b/twj8g4/93JcO)

[32. Liberzon A, Birger C, Thorvaldsdóttir H, Ghandi M, Mesirov JP, Tamayo P. The Molecular Signatures Database (MSigDB) hallmark gene set collection. Cell Syst. 2015;1:417–25.](http://paperpile.com/b/twj8g4/e1s1)

[33. Liberzon A, Subramanian A, Pinchback R, Thorvaldsdóttir H, Tamayo P, Mesirov JP. Molecular signatures database (MSigDB) 3.0. Bioinformatics. 2011;27:1739–40.](http://paperpile.com/b/twj8g4/PKrn)

[34. Jiménez-Sánchez A, Cast O, Miller ML. Comprehensive Benchmarking and Integration of Tumor Microenvironment Cell Estimation Methods. Cancer Res. 2019;79:6238–46.](http://paperpile.com/b/twj8g4/Aw52F)

[35. Aran D, Hu Z, Butte AJ. xCell: digitally portraying the tissue cellular heterogeneity landscape. Genome Biol. 2017;18:220.](http://paperpile.com/b/twj8g4/3vOhb)

[36. Yoshihara K, Shahmoradgoli M, Martínez E, Vegesna R, Kim H, Torres-Garcia W, et al. Inferring tumour purity and stromal and immune cell admixture from expression data. Nat Commun. 2013;4:2612.](http://paperpile.com/b/twj8g4/vrW2)

[37. Mao X, Xu J, Wang W, Liang C, Hua J, Liu J, et al. Crosstalk between cancer-associated fibroblasts and immune cells in the tumor microenvironment: new findings and future perspectives. Molecular Cancer. 2021;20:1–30.](http://paperpile.com/b/twj8g4/eaSlg)

[38. Yin Z, Song Y, Wang L. Single-cell RNA sequencing reveals the landscape of the cellular ecosystem of primary hepatocellular carcinoma. Cancer Cell Int. 2024;24:379.](http://paperpile.com/b/twj8g4/lPgfp)

[39. Li J, Chen H, Bai L, Tang H. Identification of CD8 T-cell exhaustion signatures for prognosis in HBV-related hepatocellular carcinoma patients by integrated analysis of single-cell and bulk RNA-sequencing. BMC Cancer. 2024;24:53.](http://paperpile.com/b/twj8g4/MUW6V)

[40. Casado P, Rodriguez-Prados J-C, Cosulich SC, Guichard S, Vanhaesebroeck B, Joel S, et al. Kinase-substrate enrichment analysis provides insights into the heterogeneity of signaling pathway activation in leukemia cells. Sci Signal. 2013;6:rs6.](http://paperpile.com/b/twj8g4/xTvSp)

[41. Wiredja DD, Koyutürk M, Chance MR. The KSEA App: a web-based tool for kinase activity inference from quantitative phosphoproteomics. Bioinformatics. 2017;33:3489–91.](http://paperpile.com/b/twj8g4/n45GM)

[42. Wilkerson MD, Hayes DN. ConsensusClusterPlus: a class discovery tool with confidence assessments and item tracking. Bioinformatics. 2010;26:1572–3.](http://paperpile.com/b/twj8g4/731Ab)

[43. Chen EY, Tan CM, Kou Y, Duan Q, Wang Z, Meirelles GV, et al. Enrichr: interactive and collaborative HTML5 gene list enrichment analysis tool. BMC Bioinformatics. 2013;14:1–14.](http://paperpile.com/b/twj8g4/2JlGs)

[44. Hu W, Shi Y, Han T, Liu C, Cao X, Shi G, et al. A Panel of E2F Target Gene Signature Predicting the Prognosis of Hepatocellular Carcinoma. Front Genet. 2022;13:879299.](http://paperpile.com/b/twj8g4/gROU7)

[45. Zhu L-J, Pan Y, Chen X-Y, Hou P-F. BUB1 promotes proliferation of liver cancer cells by activating SMAD2 phosphorylation. Oncol Lett. 2020;19:3506–12.](http://paperpile.com/b/twj8g4/inJ5z)

[46. Li Q, Qiu J, Yang H, Sun G, Hu Y, Zhu D, et al. Kinesin family member 15 promotes cancer stem cell phenotype and malignancy via reactive oxygen species imbalance in hepatocellular carcinoma. Cancer Lett. 2020;482:112–25.](http://paperpile.com/b/twj8g4/zHv7s)

[47. Zhang L, Zhuge Y, Ni J. BUB1 serves as a biomarker for poor prognosis in liver hepatocellular carcinoma. BMC Immunol. 2025;26:20.](http://paperpile.com/b/twj8g4/Lf7yo)

[48. Liu X, Zhang F, Fan Y, Qiu C, Wang K. MCM4 potentiates evasion of hepatocellular carcinoma from sorafenib-induced ferroptosis through Nrf2 signaling pathway. Int Immunopharmacol. 2024;142:113107.](http://paperpile.com/b/twj8g4/l8rVA)
